# Worldwide prevalence of haemorrhoids: a systematic review and meta-analysis

**DOI:** 10.1080/07853890.2025.2606433

**Published:** 2025-12-26

**Authors:** Amin Esmaeilnia Shirvani, Kimia Pakdaman, Zahra Maleki, Soroush Soraneh, Fatemeh Rezaei chegini, Kasra Pakdaman, Mohebat Vali, Hossein-Ali Nikbakht, Layla Shojaie, Pouyan Ebrahimi

**Affiliations:** ^a^ School of Medicine, Babol University of Medical Sciences, Babol, Iran; ^b^ Cellular and Molecular Biology Research Center, Health Research Institute, Babol University of Medical Sciences, Babol, Iran; ^c^ Shiraz University of Medical Sciences, Shiraz, Iran; ^d^ School of Nursing and Midwifery, Mazandaran University of Medical Sciences, Sari, Iran; ^e^ School of Health, Shiraz University of Medical Sciences, Shiraz, Iran; ^f^ Social Determinants of Health Research Center, Health Research Institute, Babol University of Medical Sciences, Babol, Iran; ^g^ Division of GI/Liver, Department of Medicine, Keck school of Medicine, University of Southern California, Los Angeles, CA, USA; ^h^ Cancer Research Center, Health Research Institute, Babol University of Medical Sciences, Babol, Iran

**Keywords:** Haemorrhoids, prevalence, haemorrhoid, epidemiology, meta-analysis

## Abstract

**Background:**

Haemorrhoidal disease (HD) is one of the most common anorectal disorders globally, significantly impacting individuals’ quality of life and productivity. Despite its importance, global prevalence remains unclear due to limited population-specific studies. This study aimed to systematically assess the global prevalence of HD through a systematic review and meta-analysis.

**Methods:**

We conducted a systematic review and meta-analysis by searching PubMed, Scopus, Embase, Web of Science, and Google Scholar up to March 31, 2025, without language restrictions. Studies reporting prevalence of haemorrhoids in general, clinical, or high-risk populations were included. Exclusion criteria comprised studies lacking total sample size, focusing on other anorectal conditions, or using duplicate or insufficient data. Four independent reviewers extracted and appraised study quality using the Joanna Briggs Institute tool. The primary outcome was pooled point prevalence of HD, analyzed using a random-effects model with 95% confidence intervals (CIs). The study was registered in PROSPERO (CRD420251045600).

**Results:**

From 6,312 records, 150 studies (210 datasets) comprising 8,960,338 individuals were included. The global pooled point prevalence was 25.92% (95% CI: 22.62–29.22). Lifetime prevalence was 27.19% (95% CI: 14.77–39.60), and one-year prevalence was 21.65% (95% CI: 14.33–28.97). Prevalence was higher in women (27.33%, 95% CI: 21.84–32.82) than in men, and highest in the African region 28.07% (95% CI: 15.34–40.79). Invasive diagnostic methods (28.05%, 95% CI: 23.86–32.26) yielded higher prevalence estimates than non-invasive methods. Also, factors showing associations with HD in unadjusted analyses include older age, obesity, pregnancy, diabetes, family history, constipation, and hypertension.

**Conclusion:**

HD remains a prevalent condition globally, with minor variation across regions. The burden is consistent regardless of socioeconomic context. Diagnostic method and population characteristics influence prevalence estimates. These findings underscore the importance of targeted prevention and early intervention strategies, especially for at-risk groups.

## KEY MESSAGES

1. This meta-analysis of 150 studies from 45 countries provides the most compre­hensive global estimates of haemorrhoidal disease, with pooled point, one-year, and lifetime prevalences of 25.92%, 21.65%, and 27.19%, respectively.
2. Key risk factors identified include older age, obesity, pregnancy, diabetes, constipation, and family history, all of which were associated with a higher likelihood of developing haemorrhoids.
3. The study also showed that invasive diagnostic methods generally yielded higher prevalence estimates compared to non-invasive methods, suggesting the potential for underdiagnosis in some populations.

## Introduction

Haemorrhoidal disease has been recognised since ancient times, with references in Egyptian and Greek texts [1]. Despite healthcare disparities, it remains globally prevalent. Haemorrhoids, as vascular cushions aiding continence, are classified by their position relative to the dentate line: internal (above) and external (below) [2]. Pathogenesis involves displacement of anal cushions, tissue degeneration, impaired venous return, and vascular stasis [3,4], all contributing to symptom manifestation. Symptoms occur in 40–50% of patients with haemorrhoidal disease [5]. Internal haemorrhoids commonly cause painless rectal bleeding during defecation, while external haemorrhoids are often painful due to thrombosis and perianal nerve involvement [6]. Although not life-threatening, symptoms such as bleeding, pain, and itching significantly impact quality of life and contribute to socioeconomic burdens due to absenteeism and frequent treatment needs [7]. Internal haemorrhoids are graded 1–4 by prolapse severity, though higher grades do not always correlate with symptom severity [5,8]. Risk factors include constipation-related straining, obesity, pregnancy, sedentary lifestyle, chronic diarrhea, cirrhosis with ascites, low-fibre diet, and chronic inflammatory diseases like Crohn’s [9–12]. These factors elevate venous pressure, promoting disease development, emphasising the importance of symptom assessment in understanding pathogenesis.

The true prevalence of haemorrhoidal disease is unclear due to asymptomatic cases and underreporting [4]. Diagnosis typically relies on medical history and physical examination, though procedures like colonoscopy may aid in confirmation. Reported prevalence varies widely (4.4%–38%) across studies, influenced by population characteristics, diagnostic tools, and methodologies [13–15]. Most data are from localized studies, limiting global generalizability. Therefore, a systematic review and meta-analysis is warranted to accurately assess global prevalence, enhance epidemiological understanding, and inform healthcare planning.

## Materials and methods

### Study design, search strategy and selection criteria

We report this systematic review and meta-analysis in accordance with the Preferred Reporting Items for Systematic Reviews and Meta-Analyses (PRISMA) checklist (see Table S1) [16]. The registration code for PROSPERO is CRD420251041119. We conducted a comprehensive, multivariable search across PubMed/MEDLINE, Scopus, EMBASE, and Web of Science databases from their inception until March 31, 2025. This search was performed without any restrictions on time or language. The primary search keywords included “Haemorrhoids,” “Prevalence,” and “Epidemiology” (see Table S2). Additionally, to ensure thorough coverage of all relevant studies, we also searched Google Scholar using the same key terms. Furthermore, previous systematic and review articles were examined using a similar search strategy, supplemented by the keywords “Review” and “Systematic review.” Citation tracking of prior publications was also performed to identify additional studies for further analysis.

EndNote version 2019 was used for reference management, and duplicate entries were removed using the “Duplicate Findings” tool. Four authors (Ka.P., Z.M., F.R., and L.S.) independently screened the articles based on the predefined inclusion and exclusion criteria, with disagreements resolved by the principal investigator. Eligible studies were required to report accurate data on the prevalence or incidence of haemorrhoidal disease in the studied population. Exclusion criteria included studies that: (1) enrolled patients with pre-existing haemorrhoids or those presenting with suggestive symptoms during the study period, (2) were review articles, case reports, case series, *in vitro* or *in vivo* studies, or clinical guidelines, (3) reported the prevalence of unrelated anorectal conditions (e.g. skin tags, fissures), (4) lacked total sample size information, (5) contained duplicate data, or (6) were conference abstracts without extractable data. Interventional studies were excluded if baseline prevalence data were not provided. For studies with unavailable full texts, corresponding authors were contacted *via* associated publications or the ResearchGate platform. Studies that failed to provide sufficient information or full-text access were excluded from the final analysis.

## Data extraction and variables

Data extraction was independently performed by four authors (Z.M., S.S., F.R., and A.E.S.) based on predefined inclusion and exclusion criteria, with disagreements resolved by the principal investigator (P.E.). Microsoft Excel 2019 (Microsoft, Redmond, WA, USA) was used to compile data from the selected studies. Extracted variables included the first author’s surname, publication year, data collection period, study design, population age and gender, country, diagnostic method, target population, disease onset, sample size, and number of cases. Countries were classified according to world health organization (WHO) regions [17], and income and Human Development Index (HDI) levels were determined using the latest global reports [18]. Age groups were categorized as Adult, Children, or All ages using a 19-year cutoff [19,20], with mean and standard deviation used when age ranges were not specified [21]. Diagnostic methods were grouped into Invasive (e.g. colonoscopy, sigmoidoscopy, proctoscopy) [4,22], Non-invasive (e.g. anoscopy, physical exam), and Questionnaire-based approaches [23]. Studied populations were categorized into five groups: general population, high-risk individuals, pregnant women, patients with gastrointestinal (GI) disorders, and asymptomatic patients [24–26]. Prevalence types were classified per National Institute of Mental Health guidelines as lifetime, one-year, or point prevalence [27]. Studies assessing haemorrhoid prevalence in case-control formats were extracted separately to analyze risk factors. Additionally, data on symptom types (e.g. bleeding, itching, soiling, pain, constipation), haemorrhoid type (internal/external), and grading (I–IV) were also included when available.

## Quality appraisal (risk of bias assessment)

Study quality was assessed using the Joanna Briggs Institute (JBI) Checklist for Prevalence Studies, which evaluates nine domains including sampling strategy, sample size, data analysis, statistical appropriateness, and reporting clarity. Studies scoring 5–6 were deemed moderate quality, while those scoring outside this range were classified as high or low risk [28]. Two independent reviewers conducted the quality assessments to ensure objectivity and methodological rigour. Discrepancies were resolved through discussion, with input from a team supervisor when necessary.

### Data analysis

The primary aim of this study was to estimate the prevalence of haemorrhoidal disease, with analyses conducted for three prevalence types, lifetime, one-year period, and point prevalence, stratified by gender and age group. All raw data on the number of haemorrhoid cases and total sample sizes were initially reviewed, with the main analytical focus placed on point prevalence. A random-effects meta-analysis using the restricted maximum likelihood model was applied to calculate the pooled prevalence and corresponding 95% confidence intervals (CI). Subgroup analyses were performed by WHO region, country, year of publication, data collection period, study design, diagnostic method, risk of bias, and population characteristics to evaluate potential sources of heterogeneity. Heterogeneity was assessed using Cochran’s Q test (*p* < 0.10) and quantified with the I^2^ statistic, with I^2^ values above 75% indicating substantial heterogeneity [29,30]. To further investigate heterogeneity, both univariable and multivariable meta-regression analyses were performed, incorporating variables such as publication year, data collection period (start and end year), HDI level, WHO region, income level, diagnostic method (invasive, non-invasive, or questionnaire-based), study design, age group, and target population. The multivariable model’s explanatory power was expressed as the proportion of between-study variance explained (R^2^). Sensitivity analyses were conducted by sequentially omitting each study to evaluate the stability and robustness of the pooled estimates. When significant asymmetry was detected, Duval and Tweedie’s trim-and-fill method was applied to estimate the potential influence of unpublished studies on the pooled estimates. For binary risk factors reported in both case and control groups, odds ratios (ORs) with 95% CIs were calculated. For continuous variables, standard mean differences (SMDs) were computed. All effect sizes were derived from raw data reported in the original studies and represent unadjusted estimates, as adjusted measures were not consistently available across studies. Publication bias was evaluated through funnel plots and the Egger test, with *p* < 0.05 considered statistically significant. Geographic mapping of prevalence distributions was performed using ArcGIS Pro 2.4.1 (ESRI, Redlands, CA, USA), with data categorised into six prevalence classes. All statistical analyses were carried out using STATA version 14.0 (College Station, TX, USA).

## Results

### Study selection

**Figure 1.**
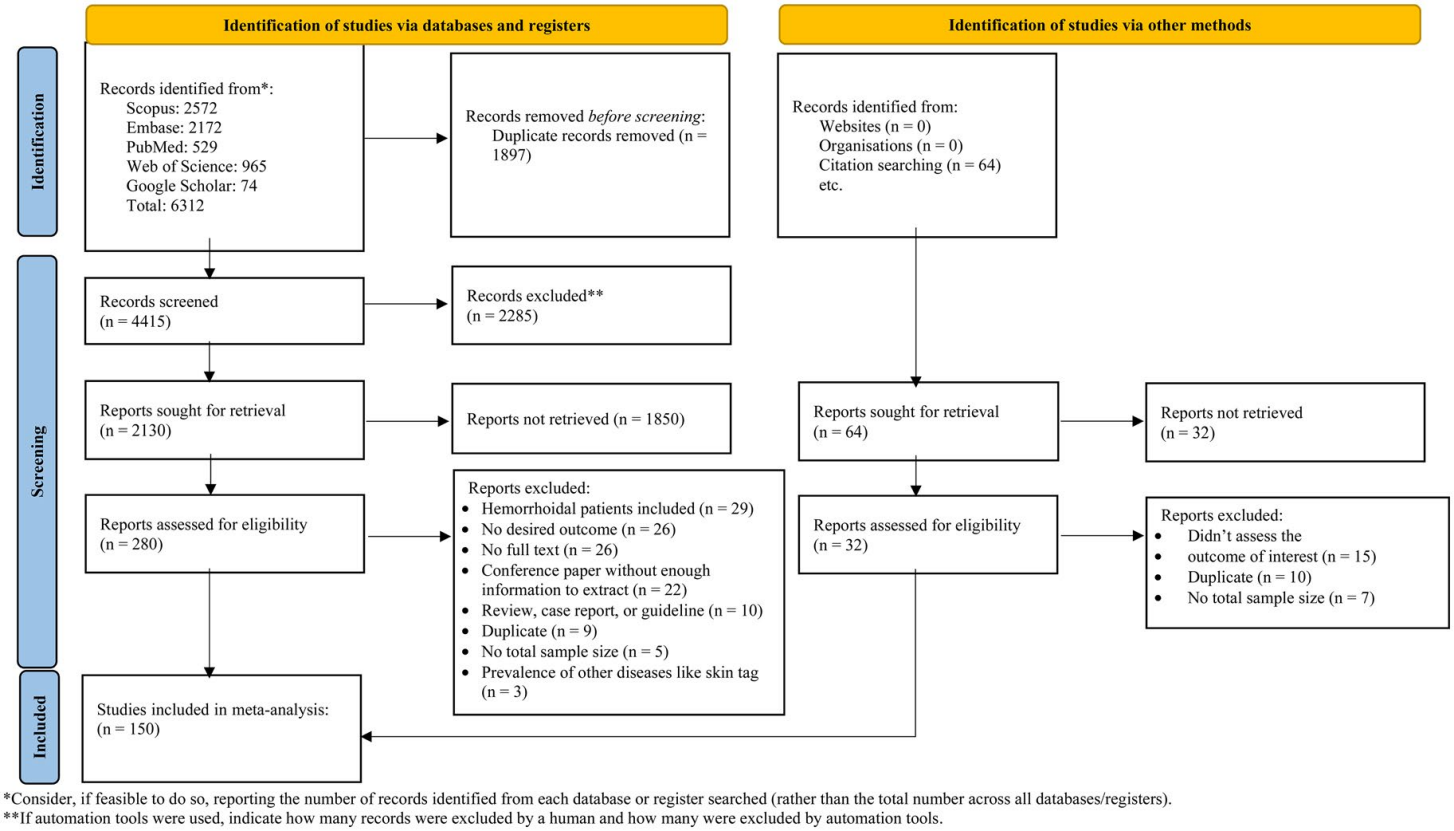
Preferred Reporting Items for Systematic Reviews and Meta-Analyses (PRISMA) flowchart – indicating the literature search strategy and the numbers of included and excluded studies.

Following the systematic search, a total of 6,312 studies were retrieved from the databases, of which 1,897 were duplicates. After the initial and secondary screening phases, 280 articles were selected for further review. Following the evaluation of these articles, a final total of 150 studies, comprising 210 records, were included for data extraction. A detailed overview of the systematic search process is presented in Figure 1.

### Characteristics of the included studies

This review included data from 8,960,338 individuals across 45 countries, providing a comprehensive overview of haemorrhoidal disease prevalence worldwide. The Region of the Americas and the European region contributed the most data, each with 28 records, while the United States alone accounted for the largest number of studies (*n* = 24). India, South Korea, and Turkey followed with 19, 8, and 7 records, respectively. The studies spanned data collection periods from 1969 to 2024 and publication years from 1970 to 2025. While five records focused on pediatric populations, the majority (*n* = 141) addressed adults. Regarding prevalence types, 163 records reported point prevalence, 25 reported lifetime prevalence, and 22 assessed one-year prevalence. Based on the Joanna Briggs Institute (JBI) criteria, risk of bias assessment categorised 25 studies as low risk, 71 as moderate risk, and 54 as high risk (see Table S3 for details).

### Global prevalence of haemorrhoids

The pooled point prevalence of haemorrhoidal disease across 163 studies was estimated at 25.92% (95% CI: 22.62–29.22), based on a total sample of 8,690,303 individuals and 1,252,259 diagnosed cases. In comparison, the one-year prevalence, calculated from 16 records with 143,492 participants and 14,155 cases, was 21.65% (95% CI: 14.33–28.97), and the lifetime prevalence, based on 15 records involving 124,818 individuals and 13,247 cases, was 27.19% (95% CI: 14.77–39.60). The analysis demonstrated considerable heterogeneity among the studies for point prevalence (*Q* = 1.8e + 06, I^2^ = 100.0%), one-year prevalence (*Q* = 5144.95, I^2^ = 99.93%), and lifetime prevalence (*Q* = 7199.10, I^2^ = 99.97%). Publication bias was assessed both visually and statistically. Funnel plot analysis and Egger’s regression test revealed no evidence of publication bias for lifetime prevalence (bias = 1.57, *p* = 0.814), but indicated significant bias for point prevalence (bias = 2.00, *p* = 0.007) and one-year prevalence (bias = 7.01, *p* = 0.001) (Figure S1). To evaluate the potential effect of this bias, Duval and Tweedie’s trim-and-fill analysis was conducted. As shown in Figure S2, no additional studies were imputed on either side of the funnel plots for point, one-year, or lifetime prevalence, and the pooled estimates remained unchanged (point: observed = 25.92%, 95% CI = 22.62–29.21; one-year: observed = 21.65%, 95% CI = 14.33–28.97; lifetime: observed = 27.19%, 95% CI = 14.77–39.60). These results suggest that, despite evidence of small-study effects, the overall pooled prevalence estimates are robust and unlikely to be substantially affected by publication bias (Figure S2).

Analysis of gender-specific data across 21 studies revealed a pooled point prevalence of haemorrhoidal disease of 25.72% (95% CI: 22.62–28.59) in males, which was lower than the prevalence in females, recorded at 27.33% (95% CI: 21.84–32.82). This gender difference was consistently observed across all timeframes assessed. Furthermore, studies reporting on pediatric populations showed a markedly lower point prevalence of haemorrhoids at 13.54% (95% CI: 4.03–23.04), approximately half the rate observed in adults, whose pooled point prevalence was estimated at 24.90% (95% CI: 21.73–28.07), as detailed in Table 1.

**Table 1.** Prevalence of haemorrhoids based on different time evaluations of studied articles.

Variables*		Point prevalence				One year prevalence				Life time prevalence			
		Number of datasets	Number of sample size / Number of haemorrhoids	Pooled Prevalence (95% CI)	Heterogeneity	Number of datasets	Number of sample size / Number of haemorrhoids	Pooled Prevalence (95% CI)	Heterogeneity	Number of datasets	Number of sample size / Number of haemorrhoids	Pooled Prevalence (95% CI)	Heterogeneity
					Q / *I*^2^				Q / *I*^2^				Q / *I*^2^
Gender	All sex	112	7611340 / 1166375	26.0 (22.30–29.70)	1.7e + 06 / 99.99	7	94190 / 6370	19.22 (7.55–30.88)	1147.61 / 99.94	13	59606 / 9128	27.81 (13.88–41.73)	7038.59 / 99.98
	Male	21	1782160 / 322256	25.72 (17.37–34.08)	6.0e + 05 / 99.99	3	44730 / 2547	11.01 (2.01–20.02)	413.50 / 99.93	6	38329 / 2169	18.51 (6.16–30.85)	379.36 / 99.91
	Female	30	2914341 / 822119	27.33 (21.84–32.82)	3797.28 / 99.99	12	99331 / 11417	21.41 (13.27–29.55)	3730.10 / 99.84	6	44509 / 3167	21.72 (9.91–33.54)	242.74 / 99.88
													
Age	All age	8	3216 / 1835	46.24 (25.46–67.02)	1640.30 / 99.52	1	417 / 20	10.96 (7.75–14.16)	–	1	365/ 40	4.80 (2.75–6.85)	–
	Adults	112	8686686 / 1250376	24.90 (21.73–28.07)	1.8e + 06 / 99.99	15	144800 / 14135	28.35 (15.23–41.46)	7196.10 / 99.98	14	124453 / 13207	22.71 (15.21–30.22)	5060.23 / 99.94
	Children	5	401 / 48	13.54 (4.03–23.04)	25.97 / 89.78	–	–	–	–	–	–	–	–

* The male population includes all studies that specifically reported the prevalence of haemorrhoids in male participants, as well as studies conducted in mixed-sex populations (All sex) that provided a male subgroup analysis. The same approach was applied for the female population. However, in the second part of the table related to age, the entire study population was categorised into three distinct groups—Children, Adults, and All age—with no overlap between them.

**Figure 2.**
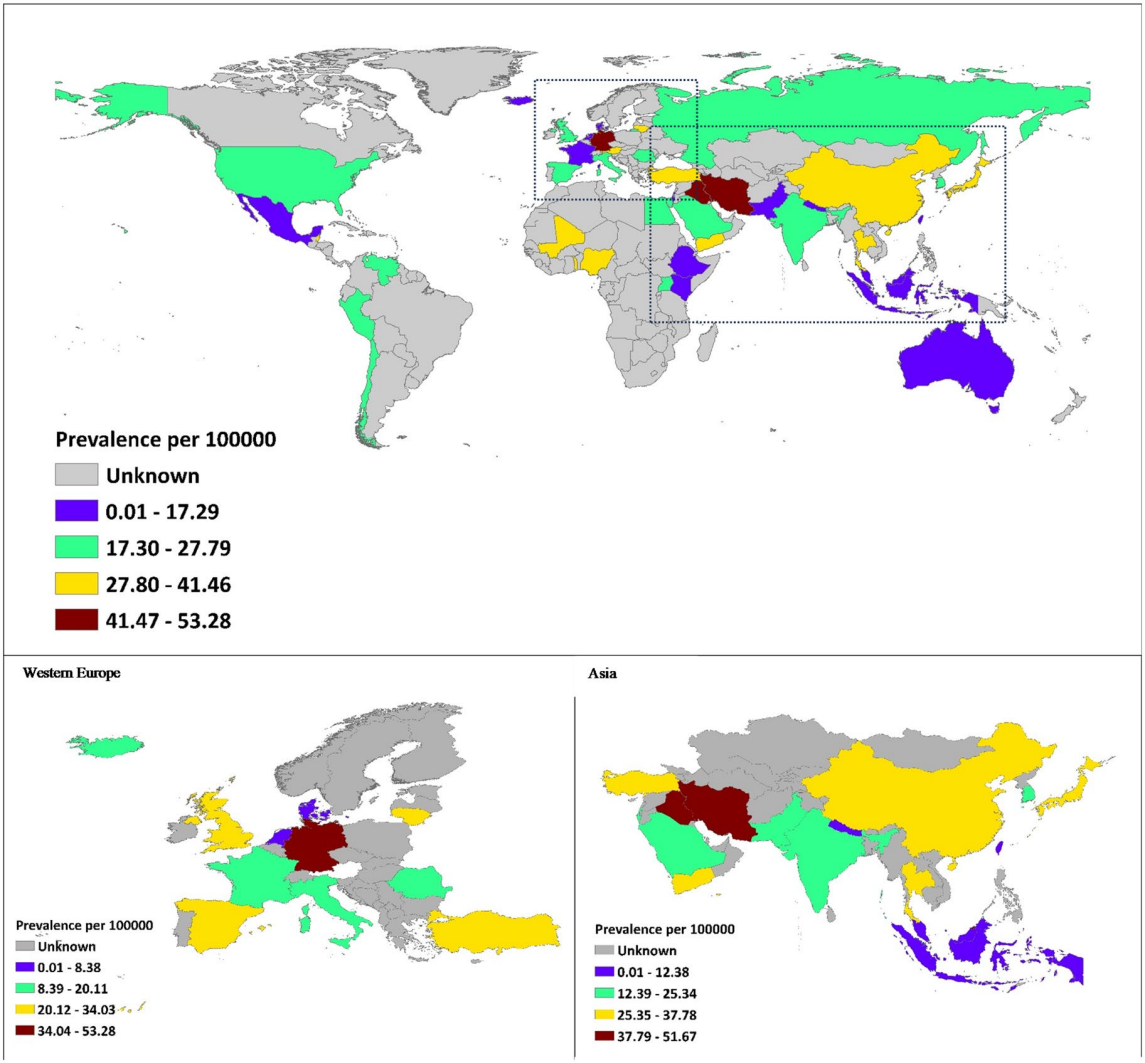
Worldwide prevalence of Haemorrhoids.

Prevalence analysis by WHO-defined regions showed that the African region had the highest pooled point prevalence of haemorrhoidal disease at 28.07% (95% CI: 15.34–40.79) based on 10 records, followed by the Region of the Americas with 26.40% (95% CI: 18.09–34.72; 28 records), and the European region at 26.03% (95% CI: 21.01–31.05; 28 records). The lowest prevalence was observed in the South-East Asia region, with 23.37% (95% CI: 17.72–29.01) from 23 records. At the country level, Germany reported the highest pooled point prevalence at 53.28% (95% CI: 49.84–56.73), followed by Iraq (51.67%, 95% CI: 42.73–60.61) and Iran (45.61%, 95% CI: 34.88–56.33), all based on single records. Conversely, Malaysia showed the lowest prevalence at 2.50% (95% CI: 0.0–5.29), followed by Kenya (4.05%, 95% CI: 1.59–6.51) and Denmark (5.92%, 95% CI: 4.38–7.47). Further details are available in Table 2 and Figure 2.

**Table 2.** Global and regional pooled point prevalence of patients with hemorrhoids.

WHO regions/ country*	Number of datasets	Number of sample size (Total)	Number of patients with hemorrhoids	Pooled Prevalence (95% CI)	Heterogeneity	
					Q	*I* ^2^
**Global**	**125**	**8690303**	**1252259**	**25.92 (22.62–29.22)**	**1.8e + 06**	**100.0**
Region of the Americas**	**28**	**3894857**	**83481**	**26.40 (18.09–34.72)**	**1.8e + 05**	**99.99**
USA	24	3892970	83093	26.60 (17.02–36.19)	1.8e + 05	99.99
Mexico	1	184	31	16.85 (11.44–22.26)	–	–
Chile	1	1399	275	19.66 (17.57–21.74)	–	–
Brazil	1	41	17	41.46 (26.38–56.54)	–	–
Venezuela	1	263	65	24.71 (19.50–29.93)	–	–
**European region**	**28**	**42367**	**10508**	**26.03 (21.01–31.05)**	**2874.72**	**99.23**
Turkey	7	1804	557	32.36 (21.21–43.51)	150.61	96.34
United Kingdom	4	29175	7716	26.50 (22.96–30.04)	16.20	87.18
Italy	4	3262	284	18.45 (5.54–31.37)	74.97	98.50
Lithuania	2	750	239	34.03 (16.29–51.77)	25.69	96.11
Iceland	1	68	9	13.24 (5.18–21.29)	–	–
Israel	1	347	60	17.29 (13.31–21.27)	–	–
Germany	1	807	430	53.28 (49.84–56.73)	–	–
Netherlands	1	1384	116	8.38 (6.92–9.84)	–	–
Peru	1	98	20	20.41 (12.43–28.39)	–	–
France	1	473	67	14.16 (11.02–17.31)	–	–
Denmark	1	895	53	5.92 (4.38–7.47)	–	–
Austria	1	967	380	38.93 (35.88–41.99)	–	–
Romania	1	1850	372	20.11 (18.28–21.93)	–	–
Russia	1	583	162	27.79 (24.15–31.42)	–	–
Spain	1	163	43	26.38 (19.61–33.15)	–	–
**South-East Asia region**	**23**	**14566**	**1893**	**23.37 (17.72–29.01)**	**630.59**	**98.28**
India	19	13919	1816	25.34 (19.46–31.21)	506.19	97.54
Nepal	2	540	55	7.44 (0.0–19.66)	39.86	97.49
Indonesia	1	62	5	8.06 (1.29–14.84)	–	–
Thailand	1	45	17	37.78 (23.61–51.94)	–	–
**Western Pacific region**	**19**	**4725833**	**1152511**	**26.31 (15.12–37.50)**	**4.3e + 05**	**100.0**
Republic of Korea	8	668615	106982	21.03 (8.76–33.29)	1366.41	99.99
China	4	2984267	960709	36.03 (4.98–67.7)	2594.11	99.99
Singapore	2	1610	517	44.09 (0.0–117.45)	1678.36	99.94
Japan	1	190	70	36.84 (29.98–43.70)	–	–
Malaysia	1	120	3	2.50 (0.0–5.29)	–	–
Brunei	1	54	26	48.15 (34.82–61.47)	–	–
Australia	1	269	16	5.95 (3.12–8.77)	–	–
Taiwan	1	1070708	84188	7.86 (7.81–7.91)	–	–
**Eastern Mediterranean Region**	**17**	**8727**	**2616**	**26.08 (17.27–34.88)**	**2117.18**	**99.20**
Pakistan	4	1305	128	16.08 (6.46–25.70)	89.13	93.93
Egypt	3	2088	462	22.51 (5.28–39.74)	110.14	98.86
Iran	3	2482	1148	45.61 (34.88–56.33)	14.68	91.59
Saudi Arabia	3	2153	626	19.53 (4.52–34.54)	80.20	97.86
Yemen	2	474	177	32.78 (0.0–89.31)	315.45	99.68
Iraq	1	120	62	51.67 (42.73–60.61)	–	–
Bahrain	1	105	13	12.38 (6.08–18.68)	–	–
**African region**	**10**	**3685**	**1250**	**28.07 (15.34–40.79)**	**1603.73**	**99.19**
Nigeria	5	1920	844	34.65 (11.94–57.37)	1270.71	99.45
Ethiopia	1	403	53	13.15 (9.85–16.45)	–	–
Kenya	1	247	10	4.05 (1.59–6.51)	–	–
Mali	1	546	192	35.16 (31.16–39.17)	–	–
Togo	1	180	56	31.11 (24.35–37.87)	–	–
Uganda	1	389	95	24.42 (20.15–28.69)	–	–

*The WHO regions are sorted based on prevalence rates, and countries are sorted based on the number of datasets.

### Stratified pooled point prevalence of haemorrhoids

Baseline variable classification revealed notable differences in the pooled point prevalence of haemorrhoidal disease across subgroups. Studies published before 2005 reported the highest prevalence at 29.31% (95% CI: 19.54–39.09). In relation to study quality, those classified as high risk of bias showed a higher pooled prevalence of 28.85% (95% CI: 20.69–37.00). Diagnostic method also influenced prevalence estimates, with invasive approaches (e.g. colonoscopy, sigmoidoscopy) yielding a higher pooled prevalence of 28.05% (95% CI: 23.86–32.26), compared to non-invasive methods at 23.03% (95% CI: 17.75–28.32). Regarding population subgroups, the highest pooled prevalence rates were observed among pregnant women (31.01%, 95% CI: 24.02–38.00) and patients with GI disorders (29.08%, 95% CI: 24.53–33.64), indicating these groups are particularly affected by haemorrhoidal disease. Further details are available in Table 3.

**Table 3.** Point prevalence estimates for patients with haemorrhoids, according to *a priori*-defined subgroups.

Variable subgroup	Number of datasets	Number of sample size (Total)	Number of patients with haemorrhoids	Pooled Prevalence (95% CI)	Heterogeneity	
					Q	*I* ^2^
**Publish year**						
Before 2005	30	9,992	2,908	29.31 (19.54–39.09)	8,101.02	99.65
2005–2015	28	407,136	87,479	24.75 (18.61–30.89)	94,375.27	99.91
2016–2025	67	8,273,175	1,161,872	24.70 (21.24–28.16)	1.6e + 06	99.99
**Start sampling date**						
Before 2000	32	305,716	32,096	28.25 (19.00–37.51)	9,097.68	99.88
2000–2015	53	5,420,784	266,041	22.72 (18.65–26.78)	3.8e + 05	99.99
2016–2025	40	2,963,803	954,122	28.17 (23.59–32.75)	8,385.27	99.51
**Type of study**						
Case-control	6	1,054	298	23.57 (7.22–39.91)	326.34	98.05
Cross-sectional	53	4,237,436	1,072,877	26.17 (21.77–30.57)	4.3e + 05	99.99
Prospective cohort	30	12,724	2,961	24.12 (18.84–29.40)	2,008.75	98.72
Retrospective cohort	36	4,439,089	176,123	27.28 (19.26–35.29)	2.9e + 05	100.0
**Risk of bias**						
High	30	7,491	2,111	28.85 (20.69–37.00)	4,749.84	99.36
Moderate	40	1,093,060	89,079	28.62 (22.15–35.10)	7,872.22	99.77
Low	35	7,583,807	1,160,136	23.50 (18.35–28.64)	1.7e + 06	100.0
**Hemorroids examination method**						
Non-invasive method	48	8,362,438	1,154,659	23.03 (17.75–28.32)	1.6e + 06	100.00
Invasive method	76	326,884	97,542	28.05 (23.86–32.26)	69,609.48	99.79
Questionare	1	981	58	5.91 (4.44–7.39)	–	–
**Population**						
General population	9	4,195,298	110,084	8.27 (4.03–12.51)	1.2e + 05	99.98
Patients (with GI problem)	72	418,971	92,923	29.08 (24.53–33.64)	1.0e + 05	99.84
High risk	6	1,191	265	18.16 (0.53–35.78)	323.50	99.00
Patients (without GI problem)	27	2,998,771	963,315	23.09 (16.66–29.52)	10,370.96	99.88
Women in pregnancy period	11	1,076,072	85,672	31.01 (24.02–38.00)	1,111.66	98.59
**Human Development Index**						
Low	14	4,828	1,450	27.39 (16.40–38.38)	2,376.23	99.33
Medium	24	15,358	2,041	22.73 (17.39–28.07)	711.89	99.39
High	24	2,992,086	963,081	30.99 (23.39–38.60)	4,558.39	99.89
Very high	63	5,678,031	285,714	24.83 (19.92–29.74)	4.1e + 05	100.0
**Income level**						
Low	5	1,603	478	28.92 (9.38–48.47)	415.31	99.15
Lower-middle	34	20,408	3410	23.74 (18.48–28.99)	2,819.66	99.14
Upper-middle	21	2,989,366	962631	33.21 (25.23–41.18)	3,106.17	99.88
High	64	5,678,926	285740	24.53 (19.66–29.39)	4.1e + 05	100.0

## Clinical hemorrhoidal information

Among the 71 studies reporting relevant clinical findings, the pooled point prevalence of symptomatic haemorrhoids was 31.22% (95% CI: 24.28–38.16), notably higher than that of asymptomatic cases, which was 21.55% (95% CI: 4.07–39.02). The prevalence of internal haemorrhoids (26.85%, 95% CI: 18.63–35.08) was approximately four times higher than that of external haemorrhoids (7.31%, 95% CI: 1.68–12.93). Rectal bleeding emerged as the most frequently reported symptom, with a pooled prevalence of 29.19% (95% CI: 23.01–35.36). In terms of clinical grading, grade II haemorrhoids were most prevalent, accounting for 16.70% (95% CI: 6.82–26.58) of cases (Table S4).

### Meta-regression analysis of point prevalence of haemorrhoids

In the univariable meta-regression analyses, a higher point prevalence of haemorrhoidal disease was observed in studies involving patients with gastrointestinal disorders (β = 7.39, 95% CI: 0.83–13.94, *p* = 0.027) and those including all-age populations (β = 21.87, 95% CI: 9.07–34.67, *p* = 0.001). In the multivariable model, after adjusting for potential confounding among covariates, these associations remained significant. Studies conducted among patients with gastrointestinal problems (β = 20.12, 95% CI: 4.15–36.08, *p* = 0.014), pregnant women (β = 24.47, 95% CI: 4.38–44.57, *p* = 0.017), and those including all-age groups (β = 18.53, 95% CI: 2.58–34.47, *p* = 0.023) demonstrated substantially higher prevalence compared with their respective reference categories. Other study-level characteristics, including study design, diagnostic method, WHO region, HDI level, and year of publication, were not significantly associated with prevalence. Overall, the multivariable model explained 16.86% of the between-study heterogeneity, suggesting that population characteristics partially accounted for the observed variability in haemorrhoid prevalence across studies (Table S5).

### Risk factors associated with haemorrhoids

Among the 150 studies, 16 focused on examining the risk factors associated with haemorrhoids between two groups: the case group (patients with haemorrhoids) and the control group (patients without haemorrhoids). Of these 16 studies, 19 variables had sufficient data for further analysis and meta-analysis. Among these 19 variables, increasing age (SMD = 0.393), obesity (OR = 1.557), pregnancy (OR = 2.606), diabetes mellitus (OR = 1.440), family history (OR = 4.192), constipation (OR = 2.508), and hypertension (OR = 1.773) were found to significantly increase the likelihood of developing haemorrhoids (further details are provided in Table 4).

**Table 4.** Risk factors associated with haemorrhoids.

Variables (number of datasets)	Number of patients without haemorrhoids / Number of patients with haemorrhoids	Effect estimate (SMD / OR)**	95% CI	P-value	Heterogeneity		
					***Q***	***I*^2^ (%)**	**P-value**
**Gender (11)**							
Male	323,959 / 64,187	OR = 1.088	0.912 − 1.298	0.348	261.22	98.29	*p* < 0.001
Female	250,989 / 45,226	Ref	–	–	–	–	–
**Age (5)**							
Case (Patients with hemorrhoids) (Mean ± SD)	52.88 ± 11.43	SMD = 0.393	0.014 − 0.771	**0.042**	92.77	94.56	*p* < 0.001
Control (Patients without hemorrhoids) (Mean ± SD)	47.66 ± 11.58	Ref	–	–	–	–	–
**BMI** (4)							
Case (Patients with hemorrhoids) (Mean ± SD)	28.57 ± 5.51	SMD = − 0.133	−0.413 − 0.147	0.352	38.64	92.94	*p* < 0.001
Control (Patients without hemorrhoids) (Mean ± SD)	28.96 ± 5.51	Ref	–	–	–	–	–
**Obesity (BMI ≥ 30) (5)**							
Yes	43,450 / 11,069	OR = 1.552	1.132 − 2.128	**0.006**	19.58	89.83	0.0017
No	133,813 / 23,137	Ref	–	–	–	–	–
**Family_status (2)**							
Married	447/ 313	OR = 2.510	0.412 − 15.31	0.319	31.51	96.83	*p* < 0.001
Single, divorced, or widowed	418/ 171	Ref	–	–	–	–	–
**Educational_status (3)**							
College and upper	513/ 199	OR = 0.899	0.577 − 1.399	0.637	5.60	67.10	0.0610
Bellow collage	814/ 559	Ref	–	–	–	–	–
**Professional status (4)**							
Employed	6,503 / 920	OR = 1.054	0.759 − 1.462	0.753	9.28	78.05	0.026
Not employed	8,954 / 1,402	Ref	–	–	–	–	–
**Pregnancy (4)**							
Yes	5,181 / 991	OR = 2.606	1.031 − 6.589	**0.043**	27.01	93.03	*p* < 0.001
No	9,707 / 1,003	Ref	–	–	–	–	–
**Cesarean delivery (2)**							
Yes	44 / 25	OR = 0.553	0.302 − 1.012	0.055	1.24	19.05	0.266
No	232 / 200	Ref	–	–	–	–	–
**Diabetes mellitus (4)**							
Yes	9,542 / 2,194	OR = 1.440	1.104 − 1.876	**0.007**	25.25	84.21	*p* < 0.001
No	167,971 / 32,304	Ref	–	–	–	–	–
**Physical activity (7)**							
High	32,492 / 6,496	OR = 1.003	0.919 − 1.095	0.949	8.46	30.30	0.207
Moderate and low	143,463 / 27,782	Ref	–	–	–	–	–
**Family history (2)**							
Yes	55 / 116	OR = 4.192	1.953 − 8.999	***p* < 0.001**	1.63	38.82	0.201
No	188 / 82	Ref	–	–	–	–	–
**Constipation (4)**							
Yes	245 / 194	OR = 2.508	1.347 − 4.667	**0.004**	13.74	74.26	0.003
No	1,939 / 1,002	Ref	–	–	–	–	–
**Hypertention (4)**							
Yes	26,013 / 5,918	OR = 1.773	1.231 − 2.551	**0.002**	72.58	95.55	*p* < 0.001
No	156,542 / 28,209	Ref	–	–	–	–	–
**Parity (2)**							
Multipara	1,752/ 471	OR = 1.150	0.720 − 1.837	0.559	2.58	2.58	0.108
Not Multipara	1,253/ 286	Ref	–	–	–	–	–
**Smoke (4)**							
Yes	80,753 / 16,638	OR = 1.126	0.875 − 1.450	0.356	8.11	84.05	0.044
No	74,176 / 15,003	Ref	–	–	–	–	–
**Multiparity (2)**							
Yes	1,281 / 471	OR = 1.150	0.720 − 1.834	0.558	2.58	61.25	0.108
No	967 / 286	Ref	–	–	–	–	–
**Fiber diet intake (4)**							
Yes	362 / 174	OR = 1.718	0.559 − 5.281	0.345	40.17	93.26	*p* < 0.001
No	2,134 / 1,079	Ref	–	–	–	–	–
**Residence status (3)**							
Urban	561 / 382	OR = 0.820	0.584 − 1.151	0.251	3.85	46.78	0.146
Rural	232 / 184	Ref	–	–	–	–	–

BMI: Body Mass Index.

** All effect sizes were calculated from raw data reported in the included studies and represent unadjusted estimates.

## Sensitivity analysis

The sensitivity analysis showed that the pooled prevalence estimates for point, one-year, and lifetime prevalence of haemorrhoidal disease remained stable when individual studies were omitted. For point prevalence, estimates ranged between 21.0% and 26.5%; for one-year prevalence, between 19.0% and 23.0%; and for lifetime prevalence, between 22.7% and 28.9%. The confidence intervals for omitted studies largely overlapped with the overall pooled estimates, indicating that no single study had a disproportionate influence on the results. These findings confirm the robustness and consistency of the pooled estimates across analyses (Figures S3, Figure S4, Figure S5).

## Discussion

This study analysed 150 studies from 45 countries, mainly from the Americas and Europe, to assess global and regional haemorrhoid prevalence. Most reported point prevalence, with fewer studies addressing lifetime and one-year prevalence. The global pooled point prevalence was 25.92%, while lifetime and one-year prevalence were 27.19% and 21.58%, respectively. The higher lifetime prevalence reflects the recurrent nature of haemorrhoidal disease [31]. Studies have reported recurrence rates of 12% and ∼5% over long-term follow-up [32,33], especially in patients with poor defecation habits [34]. Interestingly, one-year prevalence was lower than point prevalence, possibly due to reliance on self-reported questionnaires, which may miss asymptomatic or mild cases, unlike diagnostic tools such as colonoscopy and sigmoidoscopy. Additionally, many one-year prevalence studies targeted pregnant or postpartum women, reporting rates from 4% to 51% [35–39]. As pregnancy-related haemorrhoids are often transient, early cases may go unreported, limiting generalizability and potentially skewing comparisons with point prevalence.

Among the six WHO regions, the African region had the highest point prevalence of haemorrhoidal disease (28.07%), followed by the Americas (26.40%), while the lowest was in South-East Asia (23.37%). Despite regional differences, prevalence rates were relatively consistent globally. Meta-regression revealed no significant association between haemorrhoid prevalence and a country’s HDI, indicating similar disease burden regardless of healthcare disparities [40]. However, in the multivariable meta-regression model, a significant positive association was observed between haemorrhoid prevalence and the year of data collection, with prevalence increasing by approximately 1.22% per year. This finding may reflect the influence of global lifestyle changes, such as increasing sedentary behavior, aging populations, and dietary westernization, rather than improved diagnostic capacity alone. In contrast, the lack of a clear relationship with HDI suggests that haemorrhoidal disease affects populations irrespective of socioeconomic development or healthcare access. Together, these results indicate that while haemorrhoidal disease shows a modest upward temporal trend, its global distribution remains broadly consistent, driven more by biological and behavioral factors than by economic or temporal disparities. In addition, study quality should also be taken into account, as our findings indicated that studies with higher methodological quality tended to report slightly lower point prevalence estimates. Although these differences were not statistically significant when compared with studies of moderate or low quality, this trend highlights the importance of methodological rigor in prevalence research and suggests that differences in study design and diagnostic criteria may partially contribute to the variability observed across studies. The U.S. and India contributed the most data, with pooled point prevalence rates of 26.60% and 25.34%, respectively; Europe reported 26.03%. Although some countries exhibited extreme values; e.g. Malaysia (2.5%), Kenya (4%), Denmark (5.9%) vs. Germany (53.3%), Iraq (51.7%), and Brunei (48.1%), most had only a single study, limiting generalizability. A web-based survey by Sheikh et al. in European countries and Brazil estimated an overall prevalence of 11% among 16,000 respondents [41]. Reported haemorrhoid prevalence in Brazil and France was 6% and 7%, respectively, while Italy and Russia reported 16%, and other countries ranged between 9–11%. These rates are lower than those in the current study. The discrepancy may stem from the use of questionnaires in prior studies, which are limited by the high rate of asymptomatic cases [42], recall bias, and potential misclassification of other anorectal conditions, such as fissures, fistulas, pruritus, condyloma, or anal cancer, as haemorrhoids [43]. In contrast, diagnostic methods like colonoscopy offer more reliable estimates. For instance, Riss et al. reported a 38.93% prevalence in 978 Austrian patients undergoing colorectal screening, though fewer than half were symptomatic [5].

The point prevalence of haemorrhoids was slightly higher in women than in men (27.33% vs. 25.72%). This difference may be attributed to physiological and hormonal factors, particularly pregnancy, which increases intra-abdominal pressure and induces vascular changes that promote haemorrhoid development [44]. Our analysis showed that pregnancy more than doubles haemorrhoid risk. These findings are supported by prior studies reporting up to a 26% higher prevalence in women [45,46], although some studies found no significant sex differences [47,48]. Constipation, a key risk factor, is more frequent in women, especially during pregnancy and postpartum, further contributing to the disease [49,50]. Hormonal fluctuations may also affect bowel function and muscle tone. Additionally, higher healthcare-seeking behaviour among women may partly explain the increased reported prevalence [51]. Further research is warranted to clarify gender-specific mechanisms in haemorrhoidal disease.

In our study, high-risk groups, particularly pregnant women and patients with GI disorders, had the highest haemorrhoid prevalence rates (31.01% and 29.08%, respectively). The multivariable meta-regression analysis further confirmed that haemorrhoid prevalence was significantly higher among pregnant women and patients with GI disorders compared to the general population, supporting the robustness of these associations. Pregnancy significantly increases haemorrhoid risk, as confirmed by studies showing that up to one-third of women develop anorectal lesions postpartum, with nearly half being thrombosed external haemorrhoids [52]. Prevalence is notably higher postpartum than during early pregnancy [53], influenced by factors such as BMI, constipation, joint hypermobility, and infant birth weight. Mechanisms include venous stasis from uterine pressure on rectal and pelvic veins and hormonal effects (e.g. progesterone-induced reduced GI motility) [44]. Instrumental delivery raises haemorrhoid risk by increasing intra-abdominal pressure, while cesarean delivery lowers it due to the absence of pelvic strain [54,55]; however, our meta-analysis found no significant association between cesarean delivery and haemorrhoid development. Early interventions like nutritional education, constipation management, and weight control during pregnancy could reduce incidence. Patients with GI disorders are also at increased risk, due to factors like abnormal bowel habits, chronic inflammation, dietary influences, and medication use [56,57]. Managing underlying GI conditions may help prevent or alleviate haemorrhoidal disease in these populations.

Multiple studies with control groups have identified key risk factors for haemorrhoids, including obesity, pregnancy, diabetes mellitus, family history, constipation, and hypertension. Family history emerged as the strongest risk factor, increasing risk fourfold, followed by pregnancy and constipation, each doubling the risk. A meta-analysis by Kalkdijk et al. confirmed constipation’s higher prevalence among haemorrhoid patients [58]. Constipation increases intra-abdominal pressure and mechanical strain on anal cushions, contributing to haemorrhoid formation [1,15]. Family history has also been shown to significantly influence disease onset in young women [59]. Poor glycemic control in diabetic patients [60], and hypertension, potentially through vascular damage mechanisms, have also been associated with increased prevalence [46]. Supporting this, Sheikh et al. reported higher diabetes and hypertension rates in haemorrhoidpatients [41]. A Mendelian randomisation study further established obesity as a causal factor [60], with other studies confirming a higher BMI among haemorrhoid patients [5,46]. Pregnancy-related factors, including venous compression and hormonal effects, are consistent contributors [44]. While meta-regression showed no age-related trend in prevalence, haemorrhoid patients were significantly older on average, suggesting a possible age-related risk. These findings highlight the influence of physiological and lifestyle factors on haemorrhoid development, underlining the importance of preventive and early detection strategies. It should be noted that these associations should be interpreted with caution, as most included studies were cross-sectional and reported unadjusted estimates. Hence, while these factors highlight potential areas for preventive focus, causality cannot be firmly established.

While this study provides valuable global insights, several limitations should be acknowledged. High heterogeneity among included studies, arising from variations in study design, diagnostic methods, population characteristics, and data collection procedures (e.g. questionnaires, physical examinations, or colonoscopy), may affect the generalizability of the findings. Notably, almost all studies contributing to the point prevalence estimate (124 out of 125) used objectively confirmed diagnoses, with only one relying on a self-reported questionnaire. Excluding this study in sensitivity analysis did not materially change the pooled prevalence; however, such methodological variation should still be considered when interpreting the results. Although the observed heterogeneity (I^2^ > 99%) was substantial, this level of variability is common in prevalence meta-analyses encompassing data from heterogeneous populations, diagnostic methods, and study periods. Following the methodological recommendations of Barendregt et al. [61], we used a random-effects REML model, extensive subgroup analyses, and multivariable meta-regression to account for between-study variation. These approaches, along with consistent sensitivity analyses, indicate that the pooled estimates are robust and reflective of true global diversity rather than analytical inconsistency. Moreover, nearly 75% of countries lacked prevalence data, and in many others, only a single study was available, which may limit regional accuracy. Some of these single-country estimates, such as those from Germany and Malaysia, presented extreme values that should be interpreted with caution, as they may not accurately represent national-level prevalence. Although we made every effort to include all eligible and high-quality data to achieve a comprehensive global assessment, the scarcity of studies from certain regions remains a limitation. Therefore, these results should be interpreted with caution, as they may not reflect national-level estimates. Nevertheless, sensitivity analyses demonstrated that no single study exerted a disproportionate influence on the pooled global prevalence, confirming the robustness of the findings. Additionally, the meta-analysis of risk factors was based on only 16 studies, and the reported effect sizes were unadjusted, which may limit the generalizability of these associations and warrants further investigation. The use of self-reported questionnaires may also introduce recall bias and potential misclassification, particularly in asymptomatic individuals or those with other anorectal conditions. Additionally, subgroup analyses were constrained by insufficient data in certain categories, and clinical details were often incomplete. Despite these limitations, a major strength of this study lies in its rigorous systematic review and meta-analytic methodology, which yielded one of the most comprehensive global prevalence estimates for haemorrhoidal disease. The use of standardized classifications (WHO regions, HDI levels, diagnostic methods, and population subgroups) enhanced analytical precision, while the inclusion of controlled studies for risk factor assessment provided a deeper understanding of disease determinants.

## Conclusion

This systematic review and meta-analysis confirm haemorrhoidal disease as one of the most prevalent anorectal disorders worldwide, with a pooled point prevalence of approximately 26%. However, given the substantial heterogeneity and the uneven global data distribution, these estimates should be interpreted cautiously as indicative rather than definitive. The high variability among studies suggests that local prevalence may differ considerably by region, methodology, and population characteristics. Moreover, factors showing associations with haemorrhoidal disease in unadjusted analyses, including family history, obesity, constipation, pregnancy, diabetes, and hypertension, warrant further investigation in prospective studies and may inform future prevention efforts. Despite healthcare advances, haemorrhoidal disease continues to impose substantial social and economic challenges. Future research with standardised diagnostic criteria and more geographically representative data is warranted to refine global estimates and improve comparability across settings.

## Supplementary Material

Supplementary material.docx

## Data Availability

Comprehensive data can be found within the manuscript and its supplementary files. The corresponding author is available to provide further data upon a reasonable request.

## Permission to reproduce material from other sources

Not applicable, as no third-party material requiring permission was used.

## Clinical trial registration

Not applicable, as this study is a systematic review and meta-analysis and does not involve a clinical trial.
