# Supplementary material for: Worldwide prevalence of haemorrhoids: a systematic review and meta-analysis
Source: Ann Med. 2025 Dec 26;58(1):2606433. doi: 10.1080/07853890.2025.2606433 (PMC12777808; doi:10.1080/07853890.2025.2606433)
Supplement: Supplementary material.docx [file IANN_A_2606433_SM0665.docx]

**Table S1:** PRISMA Checklist

| **Section and Topic** | **Item #** | **Checklist item** | **Location where item is reported** |
| --- | --- | --- | --- |
| **TITLE** | | |  |
| Title | 1 | Identify the report as a systematic review. | 1 |
| **ABSTRACT** | | |  |
| Abstract | 2 | See the PRISMA 2020 for Abstracts checklist. | 2 |
| **INTRODUCTION** | | |  |
| Rationale | 3 | Describe the rationale for the review in the context of existing knowledge. | 2, 3 |
| Objectives | 4 | Provide an explicit statement of the objective(s) or question(s) the review addresses. | 3 |
| **METHODS** | | |  |
| Eligibility criteria | 5 | Specify the inclusion and exclusion criteria for the review and how studies were grouped for the syntheses. | 3 |
| Information sources | 6 | Specify all databases, registers, websites, organisations, reference lists and other sources searched or consulted to identify studies. Specify the date when each source was last searched or consulted. | 3 |
| Search strategy | 7 | Present the full search strategies for all databases, registers and websites, including any filters and limits used. | 3 |
| Selection process | 8 | Specify the methods used to decide whether a study met the inclusion criteria of the review, including how many reviewers screened each record and each report retrieved, whether they worked independently, and if applicable, details of automation tools used in the process. | 3 |
| Data collection process | 9 | Specify the methods used to collect data from reports, including how many reviewers collected data from each report, whether they worked independently, any processes for obtaining or confirming data from study investigators, and if applicable, details of automation tools used in the process. | 3.4 |
| Data items | 10a | List and define all outcomes for which data were sought. Specify whether all results that were compatible with each outcome domain in each study were sought (e.g. for all measures, time points, analyses), and if not, the methods used to decide which results to collect. | 4 |
|  | 10b | List and define all other variables for which data were sought (e.g. participant and intervention characteristics, funding sources). Describe any assumptions made about any missing or unclear information. | 3,4 |
| Study risk of bias assessment | 11 | Specify the methods used to assess risk of bias in the included studies, including details of the tool(s) used, how many reviewers assessed each study and whether they worked independently, and if applicable, details of automation tools used in the process. | 4 |
| Effect measures | 12 | Specify for each outcome the effect measure(s) (e.g. risk ratio, mean difference) used in the synthesis or presentation of results. | 4, 5 |
| Synthesis methods | 13a | Describe the processes used to decide which studies were eligible for each synthesis (e.g. tabulating the study intervention characteristics and comparing against the planned groups for each synthesis (item #5)). | 4 |
|  | 13b | Describe any methods required to prepare the data for presentation or synthesis, such as handling of missing summary statistics, or data conversions. | 4 |
|  | 13c | Describe any methods used to tabulate or visually display results of individual studies and syntheses. | 4 |
|  | 13d | Describe any methods used to synthesize results and provide a rationale for the choice(s). If meta-analysis was performed, describe the model(s), method(s) to identify the presence and extent of statistical heterogeneity, and software package(s) used. | 4 |
|  | 13e | Describe any methods used to explore possible causes of heterogeneity among study results (e.g. subgroup analysis, meta-regression). | 4, 5 |
|  | 13f | Describe any sensitivity analyses conducted to assess robustness of the synthesized results. | 4, 5 |
| Reporting bias assessment | 14 | Describe any methods used to assess risk of bias due to missing results in a synthesis (arising from reporting biases). | 5 |
| Certainty assessment | 15 | Describe any methods used to assess certainty (or confidence) in the body of evidence for an outcome. | 5 |
| **RESULTS** | | |  |
| Study selection | 16a | Describe the results of the search and selection process, from the number of records identified in the search to the number of studies included in the review, ideally using a flow diagram. | 6 |
|  | 16b | Cite studies that might appear to meet the inclusion criteria, but which were excluded, and explain why they were excluded. | 6, Table S2 |
| Study characteristics | 17 | Cite each included study and present its characteristics. | Table S2 |
| Risk of bias in studies | 18 | Present assessments of risk of bias for each included study. | Table S2 |
| Results of individual studies | 19 | For all outcomes, present, for each study: (a) summary statistics for each group (where appropriate) and (b) an effect estimate and its precision (e.g. confidence/credible interval), ideally using structured tables or plots. | 6,7, Tables |
| Results of syntheses | 20a | For each synthesis, briefly summarise the characteristics and risk of bias among contributing studies. | 6,7, Tables |
|  | 20b | Present results of all statistical syntheses conducted. If meta-analysis was done, present for each the summary estimate and its precision (e.g. confidence/credible interval) and measures of statistical heterogeneity. If comparing groups, describe the direction of the effect. | 6,7, 8 Tables |
|  | 20c | Present results of all investigations of possible causes of heterogeneity among study results. | 6,7, 8 Tables |
|  | 20d | Present results of all sensitivity analyses conducted to assess the robustness of the synthesized results. | Figure S3 – S5 |
| Reporting biases | 21 | Present assessments of risk of bias due to missing results (arising from reporting biases) for each synthesis assessed. | 6,7, 8 Tables |
| Certainty of evidence | 22 | Present assessments of certainty (or confidence) in the body of evidence for each outcome assessed. | 6,7, 8 Tables |
| **DISCUSSION** | | |  |
| Discussion | 23a | Provide a general interpretation of the results in the context of other evidence. | 7, 8 |
|  | 23b | Discuss any limitations of the evidence included in the review. | 9, 10 |
|  | 23c | Discuss any limitations of the review processes used. | 9, 10 |
|  | 23d | Discuss implications of the results for practice, policy, and future research. | 7 - 10 |
| **OTHER INFORMATION** | | |  |
| Registration and protocol | 24a | Provide registration information for the review, including register name and registration number, or state that the review was not registered. | 2, 3 |
|  | 24b | Indicate where the review protocol can be accessed, or state that a protocol was not prepared. | - |
|  | 24c | Describe and explain any amendments to information provided at registration or in the protocol. | - |
| Support | 25 | Describe sources of financial or non-financial support for the review, and the role of the funders or sponsors in the review. | 11 |
| Competing interests | 26 | Declare any competing interests of review authors. | 11 |
| Availability of data, code and other materials | 27 | Report which of the following are publicly available and where they can be found: template data collection forms; data extracted from included studies; data used for all analyses; analytic code; any other materials used in the review. | 11 |

*From:*  Page MJ, McKenzie JE, Bossuyt PM, Boutron I, Hoffmann TC, Mulrow CD, et al. The PRISMA 2020 statement: an updated guideline for reporting systematic reviews. BMJ 2021;372:n71. doi: 10.1136/bmj.n71. For more information, visit: <http://www.prisma-statement.org/>

**Table S2:** Search strategy

| Data base | Search strategy |
| --- | --- |
| PubMed (ALL Fields) | ((("Prevalence"[Mesh]) OR "Epidemiology"[Mesh]) OR "epidemiology" [Subheading]) AND (((("Hemorrhoids"[Mesh]) OR (Hemorrhoidal[Title/Abstract])) OR (Haemorrhoids[Title/Abstract])) OR (Haemorrhoid[Title/Abstract])) |
| Scopus (TITLE-ABS-KEY) | (("Hemorrhoids" OR "Hemorrhoid" OR "Hemorrhoidal" OR "Haemorrhoids" OR "Haemorrhoid") AND ("Prevalences" OR "Prevalence" OR "Epidemiology" OR "Epidemiologies" OR "Epidemics" OR "Incidence" OR "Morbidity" OR "Outbreaks" OR "Surveillance" OR "Endemics" OR "Occurrence" OR "Frequency")) |
| Embase | ('hemorrhoids':ab,kw,ti OR 'hemorrhoid':ab,kw,ti OR 'hemorrhoidal':ab,kw,ti OR 'haemorrhoids':ab,kw,ti OR 'haemorrhoid':ab,kw,ti) AND ('prevalences':ab,kw,ti OR 'prevalence':ab,kw,ti OR 'epidemiology':ab,kw,ti OR 'epidemiologies':ab,kw,ti OR 'epidemics':ab,kw,ti OR 'incidence':ab,kw,ti OR 'morbidity':ab,kw,ti OR 'outbreaks':ab,kw,ti OR 'surveillance':ab,kw,ti OR 'endemics':ab,kw,ti OR 'occurrence':ab,kw,ti OR 'frequency':ab,kw,ti) |
| Web of Science (Topic) | (("Hemorrhoids" OR "Hemorrhoid" OR "Hemorrhoidal" OR "Haemorrhoids" OR "Haemorrhoid") AND ("Prevalences" OR "Prevalence" OR "Epidemiology" OR "Epidemiologies" OR "Epidemics" OR "Incidence" OR "Morbidity" OR "Outbreaks" OR "Surveillance" OR "Endemics" OR "Occurrence" OR "Frequency")) |

**Table S3:** Basic information of Included Studies

| **ID** | **Author (publication year)** | **Start-date of data collection** | **End- date of data collection** | **Type of study** | **Age category** | **Gender** | **Country** | **Hemorrhoids Examination/Diagnosis** | **Population** | **Time** | **Total Sample Size (number)** | **Risk of Bias** |
| --- | --- | --- | --- | --- | --- | --- | --- | --- | --- | --- | --- | --- |
| 1 | Abasi et al. (2023) (1) | 2021 | 2021 | Cross-sectional | All age | All sex | Iran | Questionnaire | Women in pregnancy period | One year period | 417 | Low |
| 2 | Abramowitz et al. (2002) (2) | 1996 | 1997 | Prospective cohort | Adults | Female | France | Invasive method | Women in pregnancy period | One year period | 165 | Moderate |
| 3 | Abramowitz et al. (2009) (3) | 2003 | 2004 | Cross-sectional | Adults | All sex | France | Non-invasive method | High risk (HIV-patients) | Present | 473 | Low |
| 4 | Abramowitz et al. (2014) (4) | 2010 | 2010 | Retrospective cohort | Adults | All sex | France | Questionnaire | Patients | One year period | 153 | Moderate |
| 5 | Afifi et al. (2021) (5) | 2021 | 2021 | Cross-sectional | Adults | All sex | Egypt | Invasive method | Patients (with GI problem) | Present | 300 | Moderate |
| 6 | Akhtar et al. (1997) (6) | 1992 | 1996 | Retrospective cohort | Adults | All sex | USA | Invasive method | Patients (with LGBI) | Present | 100 | Conference paper |
| 7 | Alatise et al. (2012) (7) | 2007 | 2011 | Prospective cohort | Adults | All sex | Nigeria | Invasive method | Patients (with GI problem) | Present | 320 | Moderate |
| 8 | Al-Falahi et al. (2024) (8) | 2024 | 2024 | Cross-sectional | Adults | Female | Yemen | Questionnaire | General population | Life time | 400 | High |
| 9 | Al-Hadrani et al. (2000) (9) | 1995 | 1997 | Case-control | Adults | All sex | Yemen | Non-invasive method | General population / High risk (chronic khat chewers) | Present | General population = 200 / High risk = 274 | Moderate |
| 10 | Ali et al. (2012) (10) | 2008 | 2009 | Prospective cohort | Adults | All sex | Pakistan | Non-invasive method | Patients | Present | 830 | High |
| 11 | AlLehibi et al. (2024) (11) | 2010 | 2020 | Retrospective cohort | Adults | All sex | Saudi Arabia | Invasive method | Patients (with LGBI) | Present | 395 | Moderate |
| 12 | Al-Masoudi et al. (2024) (12) | 2022 | 2022 | Cross-sectional | Adults | All sex | Saudi Arabia | Questionnaire | General population | Life time | 400 | Moderate |
| 13 | Al‑Sindi et al. (2019) (13) | 2012 | 2016 | Cross-sectional | Adults | All sex | Bahrain | Invasive method | Patients | Present | 105 | Low |
| 14 | AL-Ubaide et al. (2024) (14) | 2019 | 2020 | Cross-sectional | Adults | All sex | Iraq | Invasive method | Patients (with LGBI) | Present | 120 | Moderate |
| 15 | Amir et al. (2023) (15) | 2020 | 2021 | Cross-sectional | Adults | Female | Croatia | Questionnaire | Women in pregnancy period | One year period | 1760 | Conference paper |
| 16 | Arora et al. (2012) (16) | 1995 | 2005 | Retrospective cohort | Adults | All sex | USA | Non-invasive method | Patients (with GI problem) | Present | 289681 | Low |
| 17 | Arpit et al. (2019) (17) | 2018 | 2018 | Cross-sectional | Adults | All sex | India | Invasive method | Patients (with LGBI) | Present | 232 | High |
| 18 | Aswin et al. (2019) (18) | 2018 | 2019 | Cross-sectional | Adults | All sex | India | Invasive method | Patients (with LGBI) | Present | 116 | Conference paper |
| 19 | Azhimi et al. (2023) (19) | 2016 | 2020 | Cross-sectional | Adults | All sex | Indonesia | Non-invasive method | Patients (with GI problem) | Present | 62 | Moderate |
| 20 | Bagny et al. (2021) (20) | 2020 | 2021 | Cross-sectional | Adults | All sex | Togo | Invasive method | Patients (with LGBI) | Present | 180 | Conference paper |
| 21 | Bakhshipour et al. (2023) (21) | 2011 | 2020 | Retrospective cohort | Adults | All sex | Iran | Invasive method | Patients (with LGBI) | Present | 1974 | Low |
| 22 | Balsaitiene et al. (2024) (22) | 2015 | 2019 | Prospective cohort | Adults | Female | Lithuania | Non-invasive method | Women in pregnancy period | Present | 470 | Low |
| 23 | Barles et al. (2015) (23) | 1995 | 2015 | Prospective cohort | Adults | All sex | Netherlands | Non-invasive method | Patients | Present | 1384 | Conference paper |
| 24 | Barrera et al. (2019) (24) | 2003 | 2017 | Prospective cohort | Adults | All sex | Chile | Non-invasive method | Patients | Present | 1399 | Moderate |
| 25 | Basnyat et al. (1997) (25) | 1993 | 1995 | Retrospective cohort | Adults | All sex | Nepal | Non-invasive method | General population | Present | 155 | High |
| 26 | Beksac et al. (2018) (26) | 2017 | 2018 | Cross-sectional | Adults | Female | Turkey | Non-invasive method | Women in pregnancy period | Present | 61 | High |
| 27 | Bellido et al. (2019) (27) | 2016 | 2018 | Prospective cohort | Adults | All sex | Peru | Invasive method | Patients (with LGBI) | Present | 98 | Conference paper |
| 28 | Bering et al. (2017) (28) | 2015 | 2016 | Retrospective cohort | Adults | All sex | USA | Invasive method | Patients (with GI problem) | Present | 12 | Conference paper |
| 29 | Bevan et al. (2014) (29) | 2006 | 2011 | Retrospective cohort | Adults | All sex | United Kingdom | Invasive method | Patients (with GI problem) | Present | 26251 | Low |
| 30 | Bhatti et al. (2011) (30) | 2009 | 2010 | Retrospective cohort | Adults | All sex | Pakistan | Invasive method | Patients (with LGBI) | Present | 215 | High |
| 31 | Bista et al. (2024) (31) | 2023 | 2023 | Cross-sectional | Adults | All sex | Nepal | Non-invasive method | Patients | Present | 385 | Moderate |
| 32 | Bojuwoye et al. (2021) (32) | 2013 | 2020 | Cross-sectional | Adults | All sex | Nigeria | Invasive method | Patients (with GI problem) | Present | 385 | Moderate |
| 33 | Brown et al. (1998) (33) | 1993 | 1993 | Cross-sectional | Adults | Female | Australia | Questionnaire | Women in pregnancy period | One year period | 1336 | Moderate |
| 34 | Buk et al. (2024) (34) | 2014 | 2022 | Retrospective cohort | Adults | All sex | Turkey | Non-invasive method | Patients (with GI problem) | Present | 54 | Moderate |
| 35 | Byfield et al. (1998) (35) | 1997 | 1998 | Prospective cohort | Adults | All sex | USA | Invasive method | Patients (with LGBI) | Present | 47 | Conference paper |
| 36 | Cano-Valderrama et al. (2016) (36) | 2000 | 2008 | Prospective cohort | Adults | All sex | Spain | Non-invasive method | Patients (with GI problem) | Present | 163 | Conference paper |
| 37 | Carter et al. (2013) (37) | 2005 | 2010 | Retrospective cohort | Adults | Male | Israel | Non-invasive method | Patients | Present | 347 | Low |
| 38 | Chen et al. (2023) (38) | 2009 | 2018 | Cross-sectional | Adults | Female | Taiwan | Non-invasive method | Women in pregnancy period | Present | 1070708 | Moderate |
| 39 | Chey et al. (2010) (39) | 2003 | 2008 | Prospective cohort | Adults | All sex | USA | Invasive method | General population | Present | 451 | Low |
|  |  |  |  |  |  |  |  |  | Patients (with GI problem) |  | 466 |  |
| 40 | Chiarelli et al. (2000) (40) | 1996 | 1996 | Retrospective cohort | Adults | Female | Australia | Questionnaire | General population | One year period | 40383 | Moderate |
| 41 | Choi et al. (2016) (41) | 2003 | 2015 | Prospective cohort | Adults | All sex | Republic of Korea | Non-invasive method | Patients (with GI problem) | Present | 944 | Low |
| 42 | Chong et al. (2013) (42) | 2009 | 2009 | Cross-sectional | Adults | All sex | Brunei | Invasive method | Patients (with GI problem) | Present | 54 | Moderate |
| 43 | Cirillo et al. (2009) (43) | 2005 | 2006 | Cross-sectional | Adults | All sex | Italy | Invasive method | Patients (with GI problem) | Present | 432 | Moderate |
| 44 | Coggrave et al. (2009) (44) | 2006 | 2007 | Cross-sectional | Adults | All sex | United Kingdom | Questionnaire | Patients | Life time | 1334 | High |
| 45 | Cooper et al. (2019) (45) | 2013 | 2018 | Retrospective cohort | Adults | All sex | Australia | Questionnaire | Patients | Life time | 26 | High |
| 46 | Cottrell et al. (1986) (46) | 1985 | 1985 | Prospective cohort | Adults | Female | USA | Non-invasive method | Women in pregnancy period | Present | 55 | High |
| 47 | Cristian et al. (2019) (47) | 2018 | 2019 | Cross-sectional | Adults | All sex | USA | Non-invasive method | Patients (with GIB) | Present | 83 | Conference paper |
| 48 | Cunha et al. (2020) (48) | 2020 | 2020 | Cross-sectional | Adults | Female | Brazil | Invasive method | Patients | Present | 41 | Moderate |
| 49 | Curtin et al. (2017) (49) | 2017 | 2017 | Cross-sectional | Adults | All sex | USA | Invasive method | Patients | Present | 34 | Conference paper |
| 50 | D’Alfonso et al. (2024) (50) | 2023 | 2024 | Cross-sectional | Adults | Female | Italy | Questionnaire | Women in pregnancy period | One year period | 133 | Moderate |
| 51 | Daðadóttir et al. (2024) (51) | 2010 | 2021 | Retrospective cohort | Adults | All sex | Iceland | Non-invasive method | Patients (with LGIB) | Present | 68 | Moderate |
| 52 | Dadu et al. (2022) (52) | 2022 | 2022 | Cross-sectional | Adults | All sex | India | Invasive method | Patients (with LGIB) | Present | 58 | Moderate |
| 53 | Dayal et al. (2023) (53) | 2021 | 2022 | Prospective cohort | Adults | Female | India | Invasive method | Women in pregnancy period | Present | 230 | Moderate |
| 54 | Elbadry et al. (2024) (54) | 2016 | 2021 | Retrospective cohort | Adults | All sex | Egypt | Invasive method | Patients (with GI problem) | Present | 1477 | Low |
| 55 | El-Mouzan et al. (2004) (55) | 1993 | 2002 | Retrospective cohort | Children | All sex | Saudi Arabia | Invasive method | Patients (with GI problem) | Present | 57 | High |
| 56 | Farid et al. (2022) (56) | 2020 | 2020 | Cross-sectional | Adults | Male | Malaysia | Questionnaire | High risk (Truck drivers who are exposed to prolonged sitting) | Life time | 70 | Moderate |
| 57 | Furuncuoglu et al. (1998) (57) | 1997 | 1997 | Prospective cohort | Adults | All sex | Turkey | Invasive method | Patients (with GIB) | Present | 313 | Moderate |
| 58 | Gayer et al. (2009) (58) | 1988 | 2006 | Retrospective cohort | Adults | All sex | USA | Invasive method | Patients (with LGIB) | Present | 1112 | Moderate |
| 59 | Gazet et al. (1970) (59) | 1969 | 1969 | Cross-sectional | Adults | All sex | United Kingdom | Questionnaire | Patients | Life time | 571 | High |
|  |  |  |  |  |  |  |  | Invasive method |  | Present | 39 |  |
| 60 | Ge et al. (2019) (60) | 2014 | 2014 | Retrospective cohort | Adults | All sex | China | Non-invasive method | Patients | Present | 42715 | Low |
| 61 | Gençdal et al. (2019) (61) | 2016 | 2017 | Retrospective cohort | Adults | All sex | Turkey | Invasive method | Patients | Present | 125 | High |
| 62 | Ghoshal et al. (2001) (62) | 2000 | 2001 | Case-control | Adults | All sex | India | Invasive method | Patients (with GI problem) / Patients | Present | Patients (with GI problem) = 25 / Patients = 41 | Moderate |
| 63 | Gonzalez-Ruiz et al. (2004) (63) | 1994 | 2002 | Prospective cohort | Adults | All sex | USA | Non-invasive method | High risk (Anorectal pathology in HIV/AIDS) | Present | 224 | Moderate |
| 64 | Gralnek et al. (2013) (64) | 2002 | 2008 | Retrospective cohort | Adults | All sex | USA | Invasive method | Patients (with LGIB) | Present | 76186 | Low |
| 65 | Gravante et al. (2007) (65) | 2004 | 2007 | Retrospective cohort | Adults | All sex | Italy | Invasive method | Patients (with GI problem) | Present | 160 | Low |
| 66 | Gülören et al. (2024) (66) | 2023 | 2024 | Prospective cohort | Adults | All sex | Turkey | Non-invasive method | Women in pregnancy period | Present | 51 | Low |
| 67 | Guo et al. (2024) (67) | 2017 | 2018 | Cross-sectional | Adults | All sex | China | Non-invasive method | Patients | Present | 2940295 | Low |
| 68 | Gupta et al. (2020) (68) | 2010 | 2014 | Retrospective cohort | Adults | All sex | USA | Non-invasive method | General population | Present | 3520241 | Low |
| 69 | Hajare et al. (2018) (69) | 2015 | 2015 | Cross-sectional | Adults | All sex | India | Invasive method | Patients (with LGBI) | Present | 50 | High |
| 70 | Han et al. (2009) (70) | 2002 | 2007 | Case-control | Adults | All sex | Republic of Korea | Invasive method | Patients (with GI problem) | Present | 66 | Moderate |
| 71 | Heaton et al. (1993) (71) | 1990 | 1991 | Prospective cohort | Children | All sex | United Kingdom | Invasive method | Patients (with GI problem) | Present | 60 | High |
| 72 | Herrero et al. (2001) (72) | 1998 | 1999 | Retrospective cohort | Adults | Female | Spain | Questionnaire | Women in pregnancy period | One year period | 583 | Moderate |
| 73 | Hilmi et al. (2013) (73) | 2010 | 2011 | Prospective cohort | Adults | All sex | Malaysia | Invasive method | Patients (with GI problem) | Present | 120 | Low |
| 74 | Hong et al. (2021) (74) | 2002 | 2015 | Retrospective cohort | Adults | All sex | Republic of Korea | Non-invasive method | General population | Present | 467567 | Low |
| 75 | Hong et al. (2022) (75) | 2011 | 2017 | Cross-sectional | Adults | All sex | Republic of Korea | Invasive method | General population | Present | 194620 | Low |
| 76 | Huiban et al. (2019) (76) | 2017 | 2018 | Retrospective cohort | Adults | All sex | Romania | Invasive method | Patients (with LGBI) | Present | 1850 | Moderate |
| 77 | Igwe et al. (2014) (77) | 2008 | 2012 | Retrospective cohort | Adults | All sex | Nigeria | Non-invasive method | Patients | Present | 150 | High |
| 78 | Jehangiri et al. (2017) (78) | 2014 | 2015 | Cross-sectional | All age | All sex | Pakistan | Invasive method | Patients (with LGBI) | Present | 175 | High |
| 79 | Jia et al. (2005) (79) | 2004 | 2004 | Cross-sectional | Adults | All sex | China | Non-invasive method | General population | Present | 1051 | Conference paper |
| 80 | Jo et al. (2019) (80) | 2010 | 2015 | Cross-sectional | Adults | All sex | Republic of Korea | Invasive method | Patients | Present | 1525 | Low |
| 81 | Joh et al. (2009) (81) | 2008 | 2008 | Cross-sectional | Adults | All sex | Republic of Korea | Questionnaire | General population | Present | 981 | Low |
| 82 | Kadla et al. (2016) (82) | 2010 | 2012 | Cross-sectional | Adults | All sex | India | Invasive method | Patients (with GI problem) | Present | 238 | High |
| 83 | Kang et al. (1994) (83) | 1990 | 1992 | Retrospective cohort | Adults | All sex | Singapore | Non-invasive method | Patients (with GI problem) | Present | 1063 | High |
| 84 | Karnam et al. (2004) (84) | 1998 | 2000 | Prospective cohort | Adults | All sex | USA | Invasive method | Patients (with GI problem) | Present | 105 | Moderate |
| 85 | Keller et al. (2012) (85) | 2001 | 2009 | Cross-sectional | Adults | All sex | Taiwan | Non-invasive method | Patients | Life time | 37860 | Low |
| 86 | Khan et al. (2011) (86) | 2003 | 2005 | Cross-sectional | Adults | All sex | Pakistan | Non-invasive method | Patients (with GI problem) | Present | 85 | High |
| 87 | Khandelwal et al. (2020) (87) | 2017 | 2020 | Retrospective cohort | Adults | All sex | India | Invasive method | Patients (with LGBI) | Present | 961 | Conference paper |
| 88 | Kibret et al. (2021) (88) | 2020 | 2020 | Cross-sectional | Adults | All sex | Ethiopia | Non-invasive method | Patients | Present | 403 | Low |
| 89 | Kim et al. (2013) (89) | 2008 | 2010 | Cross-sectional | Adults | All sex | Republic of Korea | Invasive method | Patients | Present | 1942 | Low |
| 90 | Klemetti et al. (2011) (90) | 1997 | 2008 | Cross-sectional | Adults | Female | United Kingdom | Non-invasive method | Women in pregnancy period | Present | 2825 | Low |
| 91 | Kuehn et al. (2009) (91) | 2005 | 2005 | Cross-sectional | Adults | All sex | Germany | Non-invasive method | Patients (with GI problem) | Present | 807 | Low |
| 92 | Lee et al. (2014) (92) | 2007 | 2009 | Cross-sectional | Adults | All sex | Republic of Korea | Non-invasive method | General population | Life time | 17228 | Low |
| 93 | Lichtarowicz et al. (1987) (93) | 1986 | 1987 | Cross-sectional | All age | All sex | United Kingdom | Questionnaire | High risk (Polish migrants) | Life time | 365 | High |
| 94 | Likhtshteyn et al. (2019) (94) | 2005 | 2015 | Cross-sectional | Adults | All sex | USA | Invasive method | Patients | Present | 64 | Conference paper |
| 95 | Longo et al. (1993) (95) | 1993 | 1993 | Prospective cohort | Adults | All sex | USA | Invasive method | Patients (with GI problem) | Present | 102 | High |
| 96 | MacLennan et al. (2000) (96) | 1998 | 1998 | Cross-sectional | Adults | All sex | Australia | Questionnaire | General population | One year period | 3010 | High |
| 97 | Martínez-Galiano et al. (2019) (97) | 2017 | 2017 | Cross-sectional | Adults | Female | Spain | Questionnaire | Women in pregnancy period | One year period | 3324 | Low |
| 98 | Medkova et al. (2019) (98) | 2016 | 2017 | Prospective cohort | Adults | Female | Russia | Non-invasive method | Women in pregnancy period | Present | 583 | Conference paper |
| 99 | Misra et al. (1996) (99) | 1996 | 1996 | Cross-sectional | Adults | All sex | India | Invasive method | Patients (with GI problem) | Present | 140 | Moderate |
| 100 | Najar et al. (2018) (100) | 2015 | 2015 | Cross-sectional | Adults | All sex | India | Non-invasive method | General population | Present | 10032 | Moderate |
| 101 | Nakaji et al. (2023) (101) | 2013 | 2021 | Cross-sectional | Adults | All sex | Japan | Non-invasive method | Patients (with GI problem) | Present | 190 | Moderate |
| 102 | Nassa et al. (2016) (102) | 2016 | 2016 | Cross-sectional | Adults | All sex | Nigeria | Questionnaire | High risk (Commercial motorcyclists) | Life time | 283 | High |
| 103 | Nikpour et al. (2008) (103) | 2004 | 2007 | Prospective cohort | All age | All sex | Iran | Invasive method | Patients (with LGBI) | Present | 496 | Moderate |
| 104 | Ogutu et al. (1998) (104) | 1996 | 1997 | Cross-sectional | Adults | All sex | Kenya | Invasive method | Patients (with GIB) | Present | 247 | High |
| 105 | Oluyemi et al. (2020) (105) | 2017 | 2019 | Cross-sectional | Adults | All sex | Nigeria | Invasive method | Patients (with LGBI) | Present | 793 | High |
| 106 | Orkin et al. (1992) (106) | 1985 | 1989 | Retrospective cohort | Adults | All sex | USA | Non-invasive method | High risk (HIV-patients) | Present | 40 | High |
| 107 | Parthasarathy et al. (2015) (107) | 2012 | 2013 | Cross-sectional | Adults | All sex | Australia | Invasive method | Patients (with GI problem) | Present | 269 | Conference paper |
| 108 | Pavone et al. (2000) (108) | 1990 | 2000 | Retrospective cohort | Adults | Male | Italy | Non-invasive method | Patients | Present | 2554 | Low |
| 109 | Peery et al. (2015) (109) | 2004 | 2008 | Cross-sectional | Adults | All sex | USA | Invasive method | Patients (with GI problem) | Present | 2813 | Low |
| 110 | Petros et al. (1990) (110) | 1986 | 1988 | Retrospective cohort | Adults | All sex | USA | Non-invasive method | Patients (with GI problem) | One year period | 111 | High |
| 111 | Poskus et al. (2014) (111) | 2010 | 2011 | Prospective cohort | Adults | Female | Lithuania | Non-invasive method | Women in pregnancy period | Present | 280 | Low |
| 112 | Qin et al. (2021) (112) | 2020 | 2021 | Cross-sectional | Adults | Female | China | Questionnaire | Women in pregnancy period | One year period | 2013 | Low |
| 113 | Qiu et al. (2024) (113) | 2020 | 2020 | Cross-sectional | Adults | All sex | China | Questionnaire | High risk (Doctors) | Life time | 1227 | Moderate |
| 114 | Rai et al. (2019) (114) | 2018 | 2019 | Cross-sectional | Children | All sex | India | Invasive method | Patients (with LGBI) | Present | 167 | Conference paper |
| 115 | Ray et al. (2024) (115) | 2023 | 2023 | Cross-sectional | Adults | All sex | India | Invasive method | Patients (with LGBI) | Present | 116 | High |
| 116 | Rhee et al. (1991) (116) | 1983 | 1989 | Cross-sectional | All age | All sex | Republic of Korea | Non-invasive method | Patients (with GIB) | Present | 970 | Moderate |
| 117 | Riss et al. (2012) (117) | 2008 | 2009 | Prospective cohort | Adults | All sex | Austria | Invasive method | Patients | Present | 976 | Low |
| 118 | Robbins et al. (2021) (118) | 2001 | 2018 | Prospective cohort | All age | All sex | USA | Invasive method | Patients (with GI problem) | Present | 68 | Moderate |
| 119 | Romano et al. (2025) (119) | 2023 | 2024 | Cross-sectional | Adults | All sex | Italy | Questionnaire | High risk (athletes) | Life time | 312 | Low |
| 120 | Rouhi et al. (2011) (120) | 2001 | 2001 | Cross-sectional | Adults | Female | Iran | Questionnaire | Women in pregnancy period | One year period | 1330 | Low |
| 121 | Sahu et al. (2017) (121) | 2016 | 2017 | Prospective cohort | All age | All sex | India | Invasive method | Patients (with LGBI) | Present | 126 | Conference paper |
| 122 | Sakr et al. (2019) (122) | 2008 | 2017 | Cross-sectional | Adults | All sex | Egypt | Invasive method | Patients (with GI problem) | Present | 311 | Low |
| 123 | Sanmee et al. (2018) (123) | 2014 | 2015 | Cross-sectional | Adults | All sex | Thailand | Invasive method | Patients (with LGBI) | Present | 45 | Moderate |
| 124 | Shafieipour et al. (2023) (124) | 2020 | 2021 | Cross-sectional | Adults | All sex | Iran | Invasive method | Patients (with LGBI) | Present | 12 | High |
| 125 | Sharma et al. (2019) (125) | 2012 | 2017 | Cross-sectional | Adults | All sex | India | Invasive method | Patients (with LGBI) | Present | 57 | Moderate |
| 126 | Sharma et al. (2022) (126) | 2019 | 2021 | Cross-sectional | Children | All sex | India | Invasive method | Patients (with LGBI) | Present | 60 | Conference paper |
| 127 | Sheikh et al. (2020) (127) | 2017 | 2018 | Cross-sectional | Children | All sex | National (Brazil, the Czech Republic, France, Hungary, Italy, Romania, Russia and Spain) | Questionnaire | General population | One year period | 16015 | Low |
| 128 | Silva et al. (2009) (128) | 2008 | 2009 | Cross-sectional | Adults | Female | Venezuela | Non-invasive method | Women in pregnancy period | Present | 263 | Conference paper |
| 129 | Singh et al. (1996) (129) | 1991 | 1992 | Cross-sectional | Adults | All sex | India | Non-invasive method | Patients | Present | 1070 | High |
| 130 | Tade et al. (2004) (130) | 1999 | 1999 | Retrospective cohort | Adults | All sex | Nigeria | Invasive method | Patients | Present | 272 | High |
| 131 | Tallarita et al. (2010) (131) | 2005 | 2008 | Prospective cohort | Adults | All sex | Italy | Non-invasive method | Patients | Present | 116 | High |
| 132 | Togo et al. (2024) (132) | 2019 | 2020 | Prospective cohort | All age | Female | Mali | Non-invasive method | Women in pregnancy period | Present | 546 | Moderate |
| 133 | Vardhan et al. (2024) (133) | 2021 | 2022 | Cross-sectional | Adults | All sex | India | Non-invasive method | Patients (with LGBI) | Present | 100 | High |
| 134 | Varma et al. (2023) (134) | 2020 | 2023 | Cross-sectional | Adults | All sex | India | Invasive method | Patients (with LGBI) | Present | 100 | High |
| 135 | Vecchia et al. (1988) (135) | 1983 | 1983 | Retrospective cohort | Adults | All sex | Italy | Questionnaire | General population | One year period | 72284 | High |
| 136 | Vecchia et al. (1992) (136) | 1983 | 1983 | Retrospective cohort | Adults | All sex | Italy | Questionnaire | General population | Life time | 63859 | Low |
| 137 | Yuhan et al. (1998) (137) | 1993 | 1997 | Retrospective cohort | Adults | All sex | USA | Non-invasive method | High risk (HIV-patients) | Present | 180 | High |
| 138 | Zacharakis et al. (2022) (138) | 2017 | 2022 | Prospective cohort | Adults | All sex | Saudi Arabia | Invasive method | Patients (with GI problem) | Present | 1701 | Low |
| 139 | Zaman et al. (1999) (139) | 1995 | 1997 | Retrospective cohort | Adults | All sex | USA | Invasive method | Patients | Present | 71 | Moderate |
| 140 | Tan et al. (2004) (140) | 1995 | 1996 | Retrospective cohort | Adults | All sex | Singapore | Invasive method | Patients (with LGBI) | Present | 547 | High |
| 141 | Wang et al. (1992) (141) | 1989 | 1990 | Retrospective cohort | Adults | All sex | China | Invasive method | Patients (with GI problem) | Present | 206 | High |
| 142 | Hass et al. (1983) (142) | 1979 | 1980 | Retrospective cohort | All age | All sex | USA | Invasive method / Non-invasive method | Patients (with GI problem) / Patients | Present | Patients (with GI problem) = 594 / Patients = 241 | Moderate |
| 143 | Mashbari et al. (2025) (143) | 2023 | 2023 | Cross-sectional | Adults | All sex | Saudi Arabia | Questionnaire | General population | One year period | 475 | Moderate |
| 144 | Ciftel et al. (2025) (144) | 2021 | 2024 | Cross-sectional | Adults | All sex | Turkey | Invasive method | Patients | Present | 752 | Low |
| 145 | Alghareeb et al. (2025) (145) | 2021 | 2024 | Retrospective cohort | Adults | All sex | Saudi Arabia | Questionnaire | General population | Life time | 148 | High |
| 146 | Mahassadi et al. (2025) (146) | 2023 | 2023 | Cross-sectional | Adults | All sex | Côte d’Ivoire | Questionnaire | General population | Life time | 735 | Low |
| 147 | Hepşen et al. (2025) (147) | 2016 | 2024 | Case-control | Adults | All sex | Turkey | Invasive method | Patients (with GI problem) | Present | 448 | Low |
| 148 | Aviles et al. (2025) (148) | 2024 | 2025 | Retrospective cohort | Adults | All sex | Mexico | Invasive method | Patients (with GI problem) | Present | 184 | Conference paper |
| 149 | Anderesen et al. (2025) (149) | 1995 | 2018 | Retrospective cohort | Adults | All sex | Denmark | Invasive method | Patients | Present | 895 | Low |
| 150 | Mutiibwa et al. (2025) (150) | 2018 | 2024 | Retrospective cohort | Adults | All sex | Uganda | Invasive method | Patients (with GI problem) | Present | 389 | Moderate |

NA; Not applicable, SD; Standard deviation, USA; united state of America, UK; United Kingdom

**Table S4:** Hemorrhoidal Information of Included Studies

| **ID** | **Author (publication year)** | **Total Sample size** | **Symptomatic Hemorrhoids** | **Asymptomatic Hemorrhoids** | **External Hemorrhoids** | **Internal Hemorrhoids** | **Hemorrhoids Grading** | | | | **Symptoms** | | | | |
| --- | --- | --- | --- | --- | --- | --- | --- | --- | --- | --- | --- | --- | --- | --- | --- |
|  |  |  |  |  |  |  | **Grade_I** | **Grade_II** | **Grade_III** | **Grade_IY** | **Bleeding** | **Itching** | **Soiling** | **Pain** | **constipation** |
| 1 | Ciftel et al. (2025) (144) | 752 | NA | NA | 34 | 166 | NA | NA | NA | NA | NA | NA | NA | NA | NA |
| 2 | Hass et al. (1983) (142) | 241 | NA | 198 | NA | NA | 42 | 134 | 22 | 0 | NA | NA | NA | NA | NA |
|  |  | 594 | 522 | NA | NA | NA | 102 | 259 | 146 | 15 | NA | NA | NA | NA | NA |
| 3 | Tan et al. (2004) (140) | 547 | 446 | NA | NA | NA | NA | NA | NA | NA | 446 | NA | NA | NA | NA |
| 4 | Zaman et al. (1999) (139) | 71 | NA | NA | 0 | 15 | NA | NA | NA | NA | NA | NA | NA | NA | NA |
| 5 | Zacharakis et al. (2022) (138) | 1701 | NA | NA | NA | 540 | NA | NA | NA | NA | NA | NA | NA | NA | NA |
| 6 | Yuhan et al. (1998) (137) | 180 | 11 | NA | NA | NA | NA | NA | NA | NA | NA | NA | NA | NA | NA |
| 7 | Varma et al. (2023) (134) | 100 | 27 | NA | NA | NA | NA | NA | NA | NA | 27 | NA | NA | NA | 25 |
| 8 | Vardhan et al. (2024) (133) | 100 | 51 | NA | 1 | 50 | 26 | 17 | 7 | NA | 51 | NA | NA | NA | NA |
| 9 | Tallarita et al. (2010) (131) | 116 | NA | NA | NA | NA | 28 | 6 | NA | NA | NA | NA | NA | NA | NA |
| 10 | Tade et al. (2004) (130) | 272 | 10 | 0 | NA | NA | NA | NA | NA | NA | NA | NA | NA | NA | NA |
| 11 | Sharma et al. (2022) (126) | 60 | 8 | NA | NA | 8 | NA | NA | NA | NA | 8 | NA | NA | NA | NA |
| 12 | Sharma et al. (2019) (125) | 57 | 9 | NA | NA | 9 | NA | NA | NA | NA | 9 | NA | NA | NA | NA |
| 13 | Shafieipour et al. (2023) (124) | 12 | 3 | NA | NA | NA | NA | NA | NA | NA | 3 | NA | NA | NA | NA |
| 14 | Sanmee et al. (2018) (123) | 45 | 17 | NA | NA | NA | NA | NA | NA | NA | 17 | NA | NA | NA | NA |
| 15 | Sakr et al. (2019) (122) | 311 | NA | NA | 3 | 21 | NA | NA | NA | NA | NA | NA | NA | NA | NA |
| 16 | Sahu et al. (2017) (121) | 126 | 32 | NA | NA | NA | NA | NA | NA | NA | 32 | NA | NA | NA | NA |
| 17 | Robbins et al. (2021) (118) | 68 | 2 | NA | NA | NA | NA | NA | NA | NA | NA | NA | NA | NA | NA |
| 18 | Riss et al. (2012) (117) | 976 | 170 | 210 | NA | NA | 227 | 70 | 31 | 2 | 100 | 91 | 15 | 37 |  |
| 19 | Rhee et al. (1991) (116) | 970 | 586 | 0 | NA | NA | NA | NA | NA | NA | NA | NA | NA | NA | NA |
| 20 | Ray et al. (2024) (115) | 116 | 116 | NA | NA | NA | NA | NA | NA | NA | 41 | NA | NA | NA | NA |
| 21 | Rai et al. (2019) (114) | 167 | 8 | NA | NA | 8 | NA | NA | NA | NA | 8 | NA | NA | NA | NA |
| 22 | Poskus et al. (2014) (111) | 280 | NA | NA | NA | NA | NA | NA | NA | NA | NA | NA | NA | NA | 107 |
| 23 | Peery et al. (2015) (109) | 2813 | NA | NA | NA | NA | NA | NA | NA | NA | NA | NA | NA | NA | 127 |
| 24 | Oluyemi et al. (2020) (105) | 793 | 575 | NA | NA | NA | NA | NA | NA | NA | 575 | NA | NA | NA | NA |
| 25 | Ogutu et al. (1998) (104) | 247 | NA | NA | NA | NA | NA | NA | NA | NA | 10 | NA | NA | NA | NA |
| 26 | Nikpour et al. (2008) (103) | 496 | 264 | NA | NA | NA | NA | NA | NA | NA | 264 | NA | NA | NA | NA |
| 27 | Nakaji et al. (2023) (101) | 190 | NA | NA | NA | 70 | NA | NA | NA | NA | NA | NA | NA | NA | NA |
| 28 | Najar et al. (2018) (100) | 10032 | NA | NA | NA | NA | NA | NA | NA | NA | NA | NA | NA | NA | 671 |
| 29 | Longo et al. (1993) (95) | 102 | 0 | 46 | NA | NA | NA | NA | NA | NA | NA | NA | NA | NA | NA |
| 30 | Kuehn et al. (2009) (91) | 807 | NA | NA | 29 | 401 | NA | NA | NA | NA | NA | NA | NA | NA | NA |
| 31 | Kim et al. (2013) (89) | 1942 | NA | NA | NA | NA | NA | NA | NA | NA | NA | NA | NA | NA | NA |
| 32 | Kibret et al. (2021) (88) | 403 | NA | NA | NA | NA | 34 | 12 | 6 | 1 | NA | NA | NA | NA | 47 |
| 33 | Khandelwal et al. (2020) (87) | 961 | 192 | NA | NA | NA | NA | NA | NA | NA | 192 | NA | NA | NA | NA |
| 34 | Khan et al. (2011) (86) | 85 | NA | 15 | NA | NA | NA | NA | NA | NA | NA | NA | NA | NA | NA |
| 35 | Kadla et al. (2016) (82) | 238 | NA | NA | 8 | 22 | NA | NA | NA | NA | NA | NA | NA | NA | NA |
| 36 | Jehangiri et al. (2017) (78) | 175 | 39 | NA | NA | NA | NA | NA | NA | NA | 39 | NA | NA | NA | NA |
| 37 | Huiban et al. (2019) (76) | 1850 | 372 | NA | NA | NA | NA | NA | NA | NA | 372 | NA | NA | NA | NA |
| 38 | Heaton et al. (1993) (71) | 60 | 20 | 0 | NA | NA | NA | NA | NA | NA | NA | NA | NA | NA | NA |
| 39 | Hajare et al. (2018) (69) | 50 | 24 | NA | NA | 24 | 2 | 11 | 5 | 6 | 24 | NA | NA | NA | NA |
| 40 | Gülören et al. (2024) (66) | 51 | NA | NA | NA | NA | 4 | 12 | 8 | 3 | 6 | 4 |  | 10 | 13 |
| 41 | Gralnek et al. (2013) (64) | 76186 | 49057 | NA | NA | 49057 | NA | NA | NA | NA | 49057 | NA | NA | NA | NA |
| 42 | Ghoshal et al. (2001) (62) | 41 | 9 | NA | NA | NA | NA | NA | NA | NA | NA | NA | NA | NA | NA |
| 43 | Gazet et al. (1970) (59) | 39 | NA | NA | NA | NA | 7 | 6 | 2 | 0 | NA | NA | NA | NA | NA |
| 44 | Gayer et al. (2009) (58) | 1112 | 250 | NA | NA | NA | NA | NA | NA | NA | 250 | NA | NA | NA | NA |
| 45 | Furuncuoglu et al. (1998) (57) | 313 | 112 | NA | NA | NA | NA | NA | NA | NA | 112 | NA | NA | NA | NA |
| 46 | Dadu et al. (2022) (52) | 58 | 9 | NA | NA | NA | NA | NA | NA | NA | 9 | NA | NA | NA | NA |
| 47 | Daðadóttir et al. (2024) (51) | 68 | 9 | NA | NA | NA | NA | NA | NA | NA | 9 | NA | NA | NA | NA |
| 48 | Cunha et al. (2020) (48) | 41 | NA | NA | 4 | 7 | 5 | 1 | NA | 1 | 5 | 4 |  | 6 | 9 |
| 49 | Cristian et al. (2019) (47) | 83 | 83 | NA | NA | NA | NA | NA | NA | NA | 15 | NA | NA | NA | NA |
| 50 | Choi et al. (2016) (41) | 944 | 63 | NA | NA | NA | NA | NA | NA | NA | NA | NA | NA | NA | NA |
| 51 | Chen et al. (2023) (38) | 1070708 | NA | NA | NA | NA | NA | NA | NA | NA | NA | NA | NA | NA | 1578 |
| 52 | Carter et al. (2013) (37) | 347 | 11 | 49 | NA | NA | NA | NA | NA | NA | NA | NA | NA | NA | NA |
| 53 | Byfield et al. (1998) (35) | 47 | 7 | NA | NA | NA | NA | NA | NA | NA | 7 | NA | NA | NA | NA |
| 54 | Buk et al. (2024) (34) | 54 | 22 | NA | NA | NA | NA | NA | NA | NA | NA | NA | NA | NA | NA |
| 55 | Bista et al. (2024) (31) | 385 | NA | NA | 33 | 30 | 22 | 17 | 12 | 2 | 29 | 18 |  | 25 | 29 |
| 56 | Bhatti et al. (2011) (30) | 215 | 49 | NA | NA | NA | NA | NA | NA | NA | 49 | NA | NA | NA | NA |
| 57 | Bellido et al. (2019) (27) | 98 | 20 | NA | NA | NA | NA | NA | NA | NA | 20 | NA | NA | NA | NA |
| 58 | Beksac et al. (2018) (26) | 61 | 24 | NA | 24 | NA | NA | NA | NA | NA | NA | NA | NA | 4 | NA |
| 59 | Barrera et al. (2019) (24) | 1399 | 266 | NA | NA | NA | NA | NA | NA | NA | NA | NA | NA | NA | NA |
| 60 | Barles et al. (2015) (23) | 1384 | 116 | NA | NA | NA | NA | NA | NA | NA | NA | NA | NA | NA | NA |
| 61 | Bakhshipour et al. (2023) (21) | 1974 | 881 | NA | NA | NA | NA | NA | NA | NA | 881 | NA | NA | NA | NA |
| 62 | Bagny et al. (2021) (20) | 180 | 56 | NA | 23 | 33 | 6 | 6 | 44 | NA | 56 | NA | NA | NA | NA |
| 63 | Aswin et al. (2019) (18) | 116 | 45 | NA | NA | NA | NA | NA | NA | NA | 45 | NA | NA | NA | NA |
| 64 | Arpit et al. (2019) (17) | 232 | 81 | NA | NA | NA | NA | NA | NA | NA | 81 | NA | NA | NA | NA |
| 65 | Arora et al. (2012) (16) | 289681 | 28118 | NA | NA | NA | NA | NA | NA | NA | NA | NA | NA | NA | NA |
| 66 | AL-Ubaide et al. (2024) (14) | 120 | 46 | 16 | NA | NA | NA | NA | NA | NA | 62 | NA | NA | NA | 42 |
| 67 | Al‑Sindi et al. (2019) (13) | 105 | 13 | NA | NA | NA | NA | NA | NA | NA | NA | NA | NA | NA | NA |
| 68 | AlLehibi et al. (2024) (11) | 395 | 83 | NA | NA | NA | NA | NA | NA | NA | 83 | NA | NA | NA | NA |
| 69 | Alatise et al. (2012) (7) | 320 | 67 | NA | NA | NA | NA | NA | NA | NA | NA | NA | NA | NA | NA |
| 70 | Akhtar et al. (1997) (6) | 100 | 30 | NA | NA | 30 | NA | NA | NA | NA | 30 |  |  |  |  |
| 71 | Afifi et al. (2021) (5) | 300 | NA | NA | NA | 115 | NA | NA | NA | NA | NA | NA | NA | NA | NA |
| **Total datasets for evaluation** | | **72** | **52** | **10** | **11** | **19** | **12** | **12** | **10** | **9** | **37** | **4** | **1** | **5** | **10** |
| **Pooled events^*^** | | **1476263** | **83031** | **534** | **159** | **50666** | **505** | **551** | **283** | **30** | **53024** | **117** | **15** | **82** | **2648** |
| **Pooled point prevalence** | | **-** | **31.22 (24.28 – 38.16)** | **21.55 (4.07 – 39.02)** | **7.31 (1.68 – 12.93)** | **26.85 (18.63 – 35.08)** | **13.58 (8.90 – 18.26)** | **16.70 (****6.82 – 26.58)** | **10.10 (4.69 – 15.51)** | **0.78 (0.09 – 1.47)** | **29.19 (23.01 – 35.36)** | **7.41 (4.28 – 10.53)** | **1.54 (0.77 – 2.31)** | **8.05 (3.52 – 12.58)** | **17.04 (8.63 – 25.45)** |

NA; not applicable,

**Table S5:** Meta-regression to assess the effect of the different variables on point prevalence of hemorrhoids.

| **Study Variables*** | **Univariable Analyses** | | | | | | | **Multivariable Analyses** | | | | | | |
| --- | --- | --- | --- | --- | --- | --- | --- | --- | --- | --- | --- | --- | --- | --- |
|  | **Coefficient** | **95% CI** | | **P-value** | **Heterogeneity** | | | **Coefficient** | **95% CI** | | **P-value** | **Heterogeneity** | | |
|  |  | **Lower** | **Upper** |  | **R-squared (%)** | **Q** | ***I*^2^ (%)** |  | **Lower** | **Upper** |  | **R-squared (%)** | **Q** | ***I*^2^ (%)** |
| **Publication year** | -0.29 | -0.59 | 0.00 | 0.053 | 2.41 | 1.4e+06 | 99.99 | 0.25 | -1.91 | 2.41 | 0.821 | 16.86 | 51739.08 | 99.77 |
| **Start year of data collection** | -0.14 | -0.43 | 0.16 | 0.371 | 0.00 | 1.6e+06 | 99.99 | 1.22 | 0.15 | 2.30 | **0.026** |  |  |  |
| **End year of data collection** | -0.29 | -0.59 | 0.00 | 0.054 | 2.41 | 1.4e+06 | 99.99 | -1.75 | -4.20 | 0.71 | 0.163 |  |  |  |
| **HDI Value** | -0.81 | -23.03 | 21.41 | 0.943 | 0.00 | 5.6e+05 | 99.99 | 17.38 | -83.46 | 118.22 | 0.735 |  |  |  |
| **Type of study (case control)** | -2.45 | -18.08 | 13.18 | 0.759 | 0.00 | 1.8e+06 | 100.0 | 2.49 | -18.53 | 23.50 | 0.817 |  |  |  |
| **Type of study (Cross-sectional)** | 0.57 | -6.13 | 7.27 | 0.868 | 0.00 | 7.2e+05 | 99.99 | 5.91 | -4.88 | 16.69 | 0.283 |  |  |  |
| **Type of study (Retrospective cohort)** | 1.82 | -5.46 | 9.10 | 0.624 | 0.00 | 7.2e+05 | 99.99 | 7.88 | -3.06 | 18.83 | 0.158 |  |  |  |
| **WHO region (South-East Asia region)** | -2.83 | -11.40 | 5.74 | 0.518 | 0.00 | 1.8e+06 | 100.0 | -5.39 | -25.58 | 14.80 | 0.601 |  |  |  |
| **WHO region (Region of the Americas)** | 0.56 | -7.42 | 8.53 | 0.892 | 0.00 | 6.2e+05 | 99.99 | -0.50 | -14.06 | 13.06 | 0.942 |  |  |  |
| **WHO region (European Region)** | 0.47 | -7.46 | 8.40 | 0.908 | 0.00 | 1.8e+06 | 100.00 | -3.13 | -15.56 | 9.29 | 0.621 |  |  |  |
| **WHO region (Eastern Mediterranean Region)** | 0.18 | -9.47 | 9.84 | 0.970 | 0.00 | 1.8e+06 | 100.0 | -5.94 | -23.25 | 11.37 | 0.501 |  |  |  |
| **WHO region (African Region)** | 2.32 | -9.75 | 14.40 | 0.706 | 0.00 | 1.8e+06 | 100.0 | -3.93 | -29.50 | 21.65 | 0.764 |  |  |  |
| **Risk of bias (moderate)** | 2.69 | -5.14 | 10.53 | 0.500 | 0.00 | 1.7e+06 | 99.99 | -1.42 | -11.83 | 8.99 | 0.790 |  |  |  |
| **Risk of bias (High)** | 2.58 | -5.88 | 11.04 | 0.550 | 0.00 | 1.8e+06 | 100.0 | -1.75 | -13.96 | 10.45 | 0.778 |  |  |  |
| **Diagnostic method (Invasive method)** | 5.38 | -1.31 | 12.08 | 0.115 | 1.31 | 1.7e+06 | 99.99 | 3.98 | -5.30 | 13.26 | 0.401 |  |  |  |
| **Diagnostic method (Questionnaire)** | -20.17 | -56.50 | 16.15 | 0.276 | 0.14 | 1.8e+06 | 100.0 | -9.97 | -49.97 | 30.03 | 0.625 |  |  |  |
| **Population (Patients (with GI problem))** | 7.39 | 0.83 | 13.94 | **0.027** | 3.28 | 1.7e+06 | 99.99 | 20.12 | 4.15 | 36.08 | **0.014** |  |  |  |
| **Population (High risk)** | -8.19 | -23.53 | 7.15 | 0.296 | 0.06 | 1.8e+06 | 100.0 | 5.24 | -16.14 | 26.63 | 0.631 |  |  |  |
| **Population (Patients (without GI problem))** | -3.55 | -11.56 | 4.45 | 0.384 | 0.00 | 4.2e+05 | 99.99 | 11.32 | -4.86 | 27.50 | 0.170 |  |  |  |
| **Population (Women in pregnancy period)** | 6.12 | -5.54 | 17.77 | 0.303 | 0.00 | 1.7e+06 | 99.99 | 24.47 | 4.38 | 44.57 | **0.017** |  |  |  |
| **Age category (Children)** | -12.23 | -29.09 | 4.63 | 0.155 | 0.85 | 1.8e+06 | 100.0 | -9.20 | -32.06 | 13.67 | 0.431 |  |  |  |
| **Age category (All age)** | 21.87 | 9.07 | 34.67 | **0.001** | 8.11 | 1.8e+06 | 99.99 | 18.53 | 2.58 | 34.47 | **0.023** |  |  |  |
| **Income level (Low)** | 3.09 | -13.63 | 19.80 | 0.717 | 0.00 | 1.8e+06 | 100.0 | 16.50 | -30.53 | 63.52 | 0.492 |  |  |  |
| **Income level (lower-middle)** | -2.94 | -10.29 | 4.41 | 0.433 | 0.00 | 1.8e+06 | 100.0 | 4.22 | -25.33 | 33.78 | 0.779 |  |  |  |
| **Income level (upper-middle)** | 8.73 | -0.08 | 17.53 | 0.052 | 2.17 | 4.2e+05 | 99.99 | 11.95 | -3.69 | 27.60 | 0.134 |  |  |  |

**Abbreviations**: HDI: human development index, CI: Confidence interval.

* These variables omitted from the model because of collinearity (Reference variables): Type of study: Prospective cohort / WHO region: Western Pacific region / Risk of bias: Low / Diagnostic method: Non-invasive method / Population: General / Age category: Adults / Income levels: High

**Table S6:** Sensitivity analysis of pooled global prevalence of hemorrhoids based on leave-one-out meta-analysis

| **Prevalence** | **Number of datasets** | **Lowest Proportion** | | **Highest Proportion** | | **Δ range (%)** | **Overall P-value** |
| --- | --- | --- | --- | --- | --- | --- | --- |
|  |  | **Author (Published year)** | **Pooled prevalence after omission (%) (95% CI)** | **Author (Published year)** | **Pooled prevalence after omission (%) (95% CI)** |  |  |
| **Point prevalence** | 125 | Hass et al. (1983) (142) | 25.38 (22.22 – 28.55) | Gupta et al. (2020) (68) | 26.13 (22.84 – 29.43) | 0.75 | 0.000 |
| **One year prevalence** | 16 | D’Alfonso et al. (2024) (50) | 19.76 (13.02 – 26.51) | Abasi et al. (2023) (1) | 22.78 (15.31 – 30.25) | 3.02 | 0.000 |
| **Life time prevalence** | 15 | Nassa et al. (2016) (102) | 22.67 (13.33 – 32.01) | Alghareeb et al. (2025) (145) | 28.89 (16.04 – 41.74) | 6.22 | 0.000 |

The Δ range represents the difference between the highest and lowest pooled estimates. The consistently small Δ values (≤ 0.75% for point, 3.02% for one-year, and 6.22% for lifetime prevalence) indicate that no individual study had a substantial influence on the overall estimates. The overall p-value (0.000) confirms statistical significance of the pooled effects across studies.


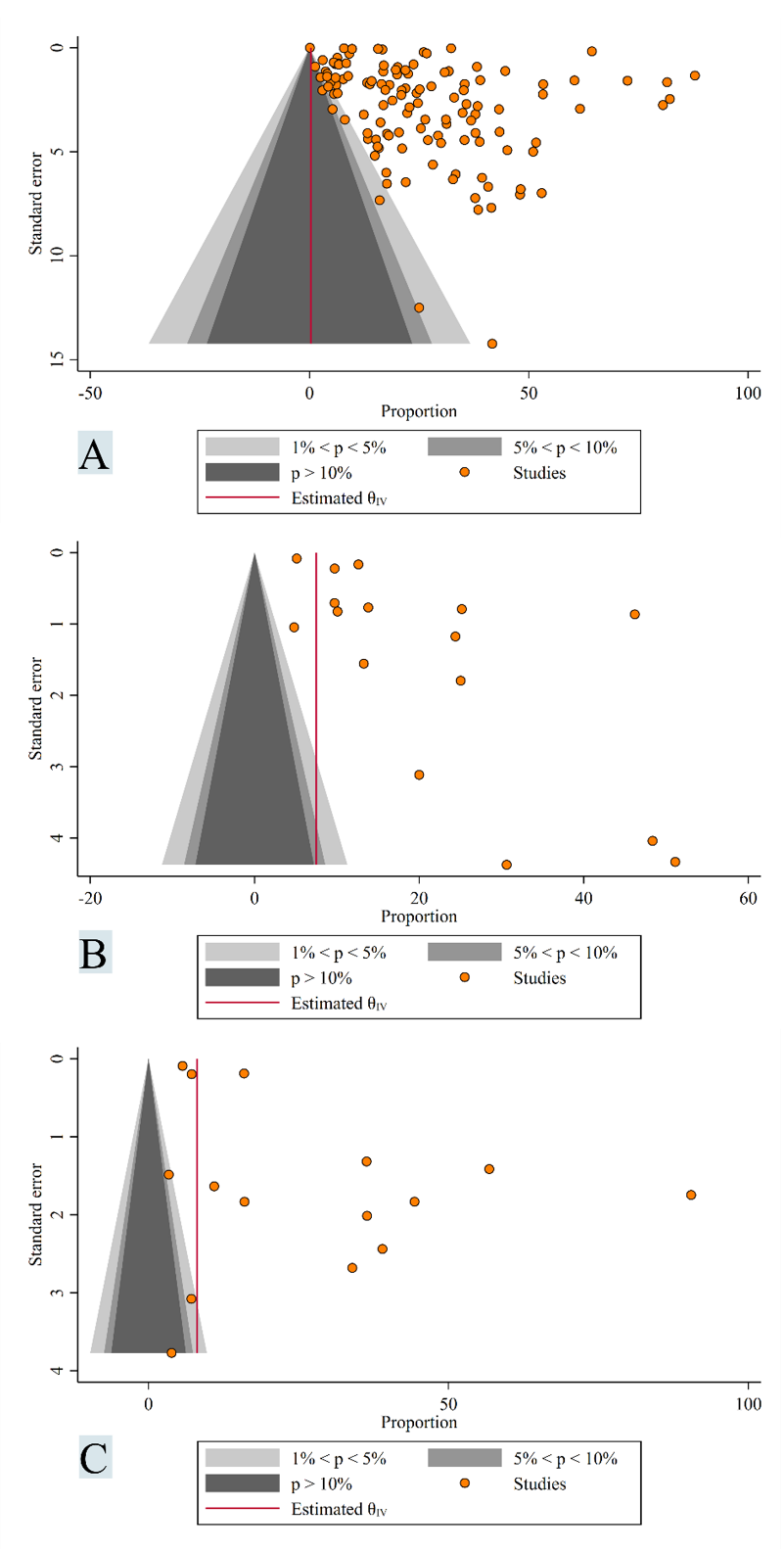


**Figure S1:** Funnel plot for the publication bias assessing of the studies in meta-analysis; A: studies assessing point prevalence of hemorrhoids, B: studies assessing one-year prevalence of hemorrhoids, and C: studies assessing life time prevalence of hemorrhoids.

**Figure S2.** Funnel plot adjusted using Duval and Tweedie’s trim-and-fill method. The red vertical line represents the estimated pooled effect size (REML method). A: studies assessing point prevalence of hemorrhoids, B: studies assessing one-year prevalence of hemorrhoids, and C: studies assessing life time prevalence of hemorrhoids.

References:

1. Abasi H, Nasrabadi FD, Gerow HJ, Yadi F, Khorashadizadeh F. The frequency of pregnancy complaints and the effective socio-demographic factors among pregnant women referring to neyshabur health centers in 2021. Middle East Journal of Rehabilitation and Health Studies. 2022;10(2).

2. Abramowitz L, Sobhani I, Benifla JL, et al. Anal fissure and thrombosed external hemorrhoids before and after delivery. Diseases of the colon & rectum. 2002;45(5):650-5.

3. Abramowitz L, Benabderrahmane D, Baron G, Walker F, Yeni P, Duval X. Systematic evaluation and description of anal pathology in HIV-infected patients during the HAART era. Diseases of the colon & rectum. 2009;52(6):1130-6.

4. Abramowitz L, Benabderrahmane M, Pospait D, Philip J, Laouénan C. The prevalence of proctological symptoms amongst patients who see general practitioners in France. The European journal of general practice. 2014;20(4):301-6.

5. Abu Aeshah WY, Afifi SAAM, Mohammed AFE, Sadek AMEM. Screening and Prevalence of Internal Hemorrhoids in patients undergoing flexible colonoscopy. The Egyptian Journal of Hospital Medicine. 2021;85(1):3474-7.

6. Akhtar AJ, Drew CR. Lower gastrointestinal hemorrhage (LGIH) in black and hispanic elderly patients. Gastrointestinal Endoscopy. 1997;45(4):AB104.

7. Alatise O, Arigbabu A, Agbakwuru E, Lawal O, Ndububa D, Ojo O. Spectrum of colonoscopy findings in Ile-Ife Nigeria. Nigerian Postgraduate Medical Journal. 2012;19(4):219-24.

8. Al-Falahi MAA. Prevalence, Risk Factors and Characteristic of Hemorrhoid among Women at Public Hospitals, Sana'a–Yemen. Al-Razi University Journal for Medical Sciences. 2024;8(1).

9. Al-Hadrani AM. Khat induced hemorrhoidal disease in Yemen. Saudi Medical Journal. 2000;21(5):475-7.

10. Ali SA, Soomro AG, Memon AI, Siddiqui AJ. Prevalence, evaluation and management of preoperative anaemia in the elective general surgical patients. Journal of Ayub Medical College Abbottabad. 2012;24(3-4):59-61.

11. AlLehibi AH, Alsubaie FF, Alzahrani RH, et al. Clinical Presentations and Risk Factors of Gastrointestinal Bleeding in the Emergency Department: A Multicenter Retrospective Study. Cureus. 2024;16(5).

12. Al-Masoudi RO, Shosho R, Alquhra D, et al. Prevalence of hemorrhoids and the associated risk factors among the general adult population in Makkah, Saudi Arabia. Cureus. 2024;16(1).

13. Al-Sindi KA, Bukhari MH, Al-Hamar MA. Role of a noninvasive stool-based molecular test in screening and early detection of colorectal cancers. Acta Medica International. 2019;6(2):82-8.

14. AL-Ubaide AF, Al-Ani RM. Bleeding Per Rectum: A Retrospective Study of 120 Cases. AL-Kindy College Medical Journal. 2024;20(1):32-6.

15. Amir L, Drandić D, Bosnjak AP, Roguljić AV, Thomson G, Zakarija-Grković I. MATERNAL PHYSICAL HEALTH AND BREASTFEEDING PROBLEMS IN CROATIA: NATIONAL ONLINE SURVEY OF NEW MOTHERS. Breastfeeding Medicine. 2023;18(5):A2-A3.

16. Arora G, Mannalithara A, Mithal A, Triadafilopoulos G, Singh G. Concurrent conditions in patients with chronic constipation: a population–based study. 2012.

17. Bansal A, Mehrotra M, Mowar A. Etiological profile of lower gastrointestinal bleed in a tertiary care hospital in northern India. 2019.

18. Aswin J, Jayakumar J, Ramkumar G, et al. Etiological profile of patients presenting with lower gastrointestinal bleeding: A tertiary center experience. Journal of Gastroenterology and Hepatology. 2019;34:794.

19. AZHIMI F, SAMPETODING S, KUSUMA MI, et al. Risk factors affecting anal fistula incidence: a single hospital study. Chirurgia. 2023;36(6):329-32.

20. Bagny A, Lawson-Ananissoh LM, Kogoe LRM, Redah D, Kaaga LY. Lower gastrointestinal bleeding in the teaching hospital campus of lome (Togo). Journal of Gastroenterology and Hepatology Research. 2021;10(1):3441-4.

21. Bakhshipour A, Rafaiee R. Upper and Lower Gastrointestinal Bleeding: A Retrospective Study on 10 Years Experiences in Southeastern Iran. Middle East Journal of Digestive Diseases. 2023;15(2):116-20.

22. Sabonyte-Balsaitiene Z, Poskus T, Jasiunas E, et al. Risk factors for constipation during pregnancy: a multicentre prospective cohort study. BMC Pregnancy Childbirth. 2024 Dec 28;24(1):878.

23. Barles AN, Bandres GP, Pera IF, Cabañuz MA, Gallizo GM, Bernadó AJ. Ambulatory anorectal surgery: Analysis of results, complications and hospital admissions. Ambulatory Surgery. 2015;21(3):114.

24. Alejandro Barrera E, Juan Riquelme C, Manuel Lizana C, Guillermo Bannura C, Adriana Zúñiga T. Elective ambulatory surgery in proctological pathology. 14 years of prospective experience in a public teaching hospital. Revista de Cirugia. 2019;71(4):293-8.

25. Basnyat B, Litch JA. Medical problems of porters and trekkers in the Nepal Himalaya. Wilderness & environmental medicine. 1997;8(2):78-81.

26. Beksac K, Aydin E, Uzelpasacı E, Akbayrak T, Ozyuncu O. Hemorrhoids and related complications in primigravid pregnancy. Journal of Coloproctology. 2018;38(3):179-82.

27. Bellido A, Espinoza-Ríos J, Pinto J, Bravo E, Gomez P, Tagle M. Independent Risk Factors for Severity and Mortality in Lower Gastrointestinal Bleeding and Proposal of New Prognosis Score. Official journal of the American College of Gastroenterology| ACG. 2019;114:S351-S2.

28. Bering J, Kahn A, Rodriguez E, Ginos B, Ramirez FC, Gurudu S. Colorectal cancer screening with cologuard in clinical practice: One-year experience and outcomes. Gastroenterology. 2017;152(5):S533.

29. Bevan R, Lee TJW, Nickerson C, Rubin G, Rees CJ, Grp NBE. Non-neoplastic findings at colonoscopy after positive faecal occult blood testing: Data from the English Bowel Cancer Screening Programme. Journal of Medical Screening. 2014;21(2):89-94.

30. Bhatti ABH, Quraishy MS. Flexible sigmoidoscopic findings in patients with fresh rectal bleeding. Journal of the College of Physicians and Surgeons Pakistan. 2011;21(9):577-8.

31. Bista CB, Chhetri ST, Kunwor B, et al. Haemorrhoids among Patients Visiting the Department of Surgery in a Tertiary Care Military Hospital of Nepal: An Observational Study. Journal of the Nepal Medical Association. 2024;62(279):765-9.

32. Bojuwoye MO, Bamidele OF, Okonkwo K, et al. The Indications and Findings at Colonoscopy in a Tertiary Hospital in North-Central Nigeria: A Seven-Year Review. West Afr J Med. 2021 Sep 30;38(9):885-91.

33. Brown S, Lumley J. Maternal health after childbirth: results of an Australian population based survey. BJOG: An International Journal of Obstetrics & Gynaecology. 1998;105(2):156-61.

34. Buk OF, Kucuk GO. A Retrospective Study of the Presentation and Management of Perianal Disease in HIV-Positive Patients Referred to a Surgical Outpatient Unit in Turkey. Med Sci Monit. 2024 Mar 26;30:e943534.

35. Byfield F, Stevens P, Finegold J, et al., editors. A prospectively validated predictive scoring system for patients with acute lower gastrointestinal bleeding. GASTROINTESTINAL ENDOSCOPY; 1998: MOSBY-YEAR BOOK INC 11830 WESTLINE INDUSTRIAL DR, ST LOUIS, MO 63146-3318 USA.

36. Cano-Valderrama O, Sanchez-Pernaute A, Rubio M, Dominguez-Serrano I, Torres A. New-onset benign anorectal disorders after bariatric surgery: Importance of bowel habit. Diseases of the Colon and Rectum. 2016;59(5):e68-e9.

37. Carter D, Levi G, Tzur D, Novis B, Avidan B. Prevalence and predictive factors for gastrointestinal pathology in young men evaluated for iron deficiency anemia. Digestive Diseases and Sciences. 2013;58(5):1299-305.

38. Chen YY, Chang CY, Lin CH, et al. Prevalence, Characteristics, and Treatment of Hemorrhoids During Pregnancy: A Nationwide Population-Based Cohort Study in Taiwan. J Womens Health (Larchmt). 2023 Dec;32(12):1394-401.

39. Chey WD, Nojkov B, Rubenstein JH, Dobhan RR, Greenson JK, Cash BD. The yield of colonoscopy in patients with non-constipated irritable bowel syndrome: Results from a prospective, controlled US trial. American Journal of Gastroenterology. 2010;105(4):859-65.

40. Chiarelli Pa, Brown W, McElduff P. Constipation in Australian women: prevalence and associated factors. International Urogynecology Journal. 2000;11:71-8.

41. Choi YS, Kim WJ, Kim JK, Song KH, Jung HJ. Incidence and clinical features of perianal diseases in patients with ulcerative colitis in Korea. Journal of Crohn's and Colitis. 2016;10:S186.

42. Chong VH, Bakar S, Sia R, et al. Colorectal Cancer Screening among Government Servants in Brunei Darussalam. Asian Pacific Journal of Cancer Prevention. 2013;14(12):7657-61.

43. Cirillo M, Pizzi AL, Gargiulo G, et al. Il ruolo della coloscopia nella quarta età. Annali Italiani di Chirurgia. 2009;80(2):131-4.

44. Coggrave M, Norton C, Wilson-Barnett J. Management of neurogenic bowel dysfunction in the community after spinal cord injury: a postal survey in the United Kingdom. Spinal cord. 2009;47(4):323-33.

45. Cooper E, Bonne Lee B, Muhlmann M. Outcomes following stoma formation in patients with spinal cord injury. Colorectal Disease. 2019;21(12):1415-20.

46. Cottrell BH, Shannahan MD. Effect of the birth chair on duration of second stage labor and maternal outcome. Nursing research. 1986;35(6):364-7.

47. Cristian T, Goldis A, Sporea I. Upper versus lower gastrointestinal bleeding. A direct comparison of clinical presentation, outcomes and the risk factors. Journal of Gastrointestinal and Liver Diseases. 2019;28:169-70.

48. Cunha E, Guzela V, Balbi G, Sobrado C, Andrade D. Anorectal diseases in patients with Antiphospholipid syndrome: a cross-sectional study. Advances in Rheumatology. 2020;60:51.

49. Curtin BF, Chitsaz E, Powers A, et al. Clinical, Endoscopic, and Histologic GI Manifestations of Behcet's Disease: Time to Redefine the Syndrome? Gastroenterology. 2017;152(5):S777.

50. D’Alfonso A, De Carolis F, Serva A, Valiyeva S, Guido M, Pietroletti R. Haemorrhoidal disease in pregnancy: results from a self-assessment questionnaire administered by means of a social network. BMC gastroenterology. 2024;24(1):150.

51. Daðadóttir SM, Ingason AB, Hreinsson JP, Björnsson ES. Comparison of gastrointestinal bleeding in patients with and without liver cirrhosis. Scandinavian Journal of Gastroenterology. 2024;59(9):1081-6.

52. Dadu F, Kumar M. Colonoscopic Profile of Lower Gastrointestinal Bleed in Adults: A Tertiary Care Center-based Study in South India. The Journal of the Association of Physicians of India. 2023;71(1):1-.

53. Dayal P, Meena KS, Jaiswal RP, Gupta AK, Chawla D, Dewanda NK. A Prospective Cohort Study of Prevalence of Anorectal Diseases during Pregnancy. International Journal of Toxicological and Pharmacological Research. 2023;13(4):264-71.

54. Elbadry M, El-Raey F, Alboraie M, et al. Clinical and endoscopic characteristics of patients undergoing gastrointestinal endoscopic procedures in Egypt: a nationwide multicenter study. BMC gastroenterology. 2024;24(1):186.

55. El-Mouzan MI, Abdullah AM. Yield of colonoscopy in children with rectal bleeding. Saudi Medical Journal. 2004;25(8):998-1001.

56. Farid MINA, Azhar ANH, Iskandar HR, Abdiweli H, Selamat HA. A cross-sectional study on the prevalence of haemorrhoids among lorry drivers in Klang valley, Malaysia. International journal of health sciences.6(S7):5153-61.

57. Furuncuoglu Y, Mungan Z, Yeginsu O, et al. Demographic, clinic and etiologic features of the patients with rectal bleeding. Turkish Journal of Gastroenterology. 1998;9(3):279-83.

58. Gayer C, Chino A, Lucas C, et al. Acute lower gastrointestinal bleeding in 1,112 patients admitted to an urban emergency medical center. Surgery. 2009;146(4):600-7.

59. Gazet JC, Redding W, Rickett JW. The prevalence of haemorrhoids. A preliminary survey. Proc R Soc Med. 1970;63 Suppl(Suppl 1):78-80.

60. Ge T, Zhang Q, Lu J, Chen G, Sun M, Li X. Association between education and health outcomes among adults with disabilities: evidence from Shanghai, China. PeerJ. 2019;7:e6382.

61. Gençdal G. Demir Eksikliği Anemisi olan Hastalarda Üst ve Alt Gastrointestinal Sistem Endoskopisi Sonuçlarımız. Gazi Medical Journal. 2019;30(1).

62. Ghoshal UC, Biswas PK, Roy G, Pal BB, Dhar K, Banerjee PK. Colonic mucosal changes in portal hypertension. Trop Gastroenterol. 2001 Jan-Mar;22(1):25-7.

63. Gonzalez-Ruiz C, Heartfield W, Briggs B, Vukasin P, Beart RW. Anorectal pathology in HIV/AIDS-infected patients has not been impacted by highly active antiretroviral therapy. Diseases of the Colon and Rectum. 2004;47(9):1483-6.

64. Gralnek IM, Fisher OR-T, Holub JL, Eisen GM. The role of colonoscopy in evaluating hematochezia: a population-based study in a large consortium of endoscopy practices. Gastrointestinal endoscopy. 2013;77(3):410-8.

65. Gravante G, Delogu D, Venditti D. Upper and lower gastrointestinal diseases in liver transplant candidates. International journal of colorectal disease. 2008;23:201-6.

66. Gülören G, Çınar GN, Baran E, et al. Hemorrhoids, Anorectal Symptoms, and Related Risk Factors in Pregnancy and the Postpartum Period: A Follow-up Study. The Journal of Women's & Pelvic Health Physical Therapy. 2024;48(3):184-93.

67. Guo C, Che X, Lin Z, et al. Epidemiological characteristics of hemorrhoids in a healthy physical examination population in China. Beijing da xue xue bao Yi xue ban= Journal of Peking University Health Sciences. 2024;56(5):815-9.

68. Gupta K, Khan A, Kumar M, Sawalha K, Abozenah M, Singhania R. Readmissions rates after myocardial infarction for gastrointestinal bleeding: a national perspective. Digestive Diseases and Sciences. 2021;66:751-9.

69. Heaton N, Davenport M, Howard E. Incidence of haemorrhoids and anorectal varices in children with portal hypertension. Journal of British Surgery. 1993;80(5):616-8.

70. Han SJ, Kim CM, Lee JE, Lee TH. Colonoscopic Lesions in Patients With Spinal Cord Injury. Journal of Spinal Cord Medicine. 2009 Aug;32(4):404-7.

71. Heaton ND, Davenport M, Howard ER. Incidence of haemorrhoids and anorectal varices in children with portal hypertension. Br J Surg. 1993 May;80(5):616-8.

72. Tosal Herrero B, Richart Martínez M, Luque Plaza M, et al. Gastrointestinal signs and symptoms during pregnancy and postpartum in a sample of Spanish women. Atencion primaria / Sociedad Española de Medicina de Familia y Comunitaria. 2001;28(1):53-8.

73. Hilmi I, Hartono JL, Pailoor J, Mahadeva S, Goh KL. Low prevalence of 'classical' microscopic colitis but evidence of microscopic inflammation in Asian Irritable Bowel Syndrome patients with diarrhoea. BMC Gastroenterology. 2013;13(1).

74. Hong J, Kim I, Song J, Ahn BK. Socio-demographic factors and lifestyle associated with symptomatic hemorrhoids: Big data analysis using the National Health insurance Service-National Health screening cohort (NHIS-HEALS) database in Korea. Asian J Surg. 2022 Jan;45(1):353-9.

75. Hong YS, Jung KU, Rampal S, et al. Risk factors for hemorrhoidal disease among healthy young and middle-aged Korean adults. Sci Rep. 2022 Jan 7;12(1):129.

76. Huiban L, Stanciu C, Muzica CM, et al. The frequency of inflammatory bowel disease as cause of rectoragia-a single center experience. Journal of Gastrointestinal and Liver Diseases. 2019;28:11.

77. Igwe PO, Dodiyi-Manuel A, Oparaku KC. The pattern of surgically treatable anorectal diseases in University of Port Harcourt Teaching Hospital, Rivers State, Nigeria. Niger J Med. 2014 Jan-Mar;23(1):57-60.

78. Jehangiri AUR, Gul R, Hadayat R, Khan AN, Zabiullah, Khursheed L. Causes Of Lower Gastrointestinal Bleeding On Colonoscopy. Journal of Ayub Medical College, Abbottabad : JAMC. 2017;29(3):468-71.

79. Jia LH, Wu CZ. Mental and body health status of doctors and nurses in a military hospital in Shenyang. Chinese Journal of Clinical Rehabilitation. 2005;9(28):86-8.

80. Jo SY, Park H, Lee BK, Baik SJ, Lee HJ, Park YM. Prevalence of and risk factors for diseases in Korean Americans and native Koreans undergoing health checkup. Korean Journal of Family Medicine. 2019;40(6):388-94.

81. Joh HK, Seong MK, Oh SW. Fecal incontinence in elderly Koreans. Journal of the American Geriatrics Society. 2010;58(1):116-21.

82. Kadla SA, Shah NA, Bindroo MA, et al. Evaluation of iron deficiency anaemia for gastrointestinal causes in patients without GI symptoms in high prevalent GI malignancy zones. Arab J Gastroenterol. 2016 Jun;17(2):67-72.

83. Kang J, Yap I, Gwee K. The pattern of functional and organic disorders in an Asian gastroenterological clinic. Journal of gastroenterology and hepatology. 1994;9(2):124-7.

84. Karnam US, Felder LR, Raskin JB. Prevalence of occult celiac disease in patients with iron-deficiency anemia: a prospective study. South Med J. 2004 Jan;97(1):30-4.

85. Keller JJ, Lin HC. Haemorrhoids are associated with erectile dysfunction: A population-based study. International Journal of Andrology. 2012;35(6):867-72.

86. Khan IM, Hassan MK, Rahman S, et al. FREQUENCY OF ORGANIC PATHOLOGIES IN PATIENTS WITH IRRITABLE BOWEL SYNDROME. Journal of Postgraduate Medical Institute. 2011 08/17;23(4).

87. Khandelwal R, Das HS, Panda C, et al. Lower gastrointestinal bleed: Experience from a tertiary care hospital in coastal Odisha. Indian Journal of Gastroenterology. 2020;39(SUPPL 1):S40.

88. Kibret AA, Oumer M, Moges AM. Prevalence and associated factors of hemorrhoids among adult patients visiting the surgical outpatient department in the University of Gondar Comprehensive Specialized Hospital, Northwest Ethiopia. PLoS One. 2021;16(4):e0249736.

89. Kim HS, Baik SJ, Kim KH, et al. Prevalence of and risk factors for gastrointestinal diseases in korean americans and native koreans undergoing screening endoscopy. Gut and Liver. 2013;7(5):539-45.

90. Klemetti R, Kurinczuk JJ, Redshaw M. Older women's pregnancy related symptoms, health and use of antenatal services. Eur J Obstet Gynecol Reprod Biol. 2011 Feb;154(2):157-62.

91. Kuehn HG, Gebbensleben O, Hilger Y, Rohde H. Relationship between anal symptoms and anal findings. International Journal of Medical Sciences. 2009;6(2):77-84.

92. Lee J-H, Kim H-E, Kang J-H, Shin J-Y, Song Y-M. Factors associated with hemorrhoids in korean adults: korean national health and nutrition examination survey. Korean journal of family medicine. 2014;35(5):227.

93. Lichtarowicz A, Mayberry J. Gastrointestinal disease amongst Polish migrants in Nottingham. Journal of the Royal Society of Medicine. 1987;80(11):692-3.

94. Arroyo-Mercado FMM, Likhtshteyn M, Alshal M, Reka S. Where can the lesion be? A pelvic GIST with both a normal upper and lower endoscopy. American Journal of Gastroenterology. 2019;114:S1142-S3.

95. Longo WE, Dean PA, Virgo KS, Vernava AM. Colonoscopy in patients with benign anorectal disease. Diseases of the colon & rectum. 1993;36:368-71.

96. MacLennan AH, Taylor AW, Wilson DH, Wilson D. The prevalence of pelvic floor disorders and their relationship to gender, age, parity and mode of delivery. BJOG: An International Journal of Obstetrics & Gynaecology. 2000;107(12):1460-70.

97. Martínez-Galiano JM, Delgado-Rodríguez M, Rodríguez-Almagro J, Hernández-Martínez A. Symptoms of discomfort and problems associated with mode of delivery during the puerperium: An observational study. International Journal of Environmental Research and Public Health. 2019;16(22).

98. Medkova Y, Markarian D, Tulina I, et al. Haemorrhoids during pregnancy and after childbirth: An observational study. Colorectal Disease. 2019;21:128.

99. Misra SP, Dwivedi M, Misra Y. Prevalence and factors influencing hemorrhoids, anorectal varices, and colopathy in patients with portal hypertension. Endoscopy. 1996;28(4):340-5.

100. FA N, Faisal M, Khesal A, Ansari T. Prevalence of hemorrhoid among the patients visiting surgery OPD at Nium hospital. European Journal of Biomedical. 2018;5(1):435-7.

101. Nakaji K, Kumamoto M, Okahara K, et al. Usefulness and safety of observing anorectal lesions by colon capsule endoscopy. Journal of Japanese Society of Gastroenterology. 2023;120(11):912-9.

102. Nassa YG, Danjuma A, Ayuba SB, Yahaya SA, Inusa B, Yakubu I. Prevalence and predictors of hemorrhoids among commercial motorcyclists in Kaduna state, Nigeria. World Journal of Preventive Medicine. 2016;4(1):1-4.

103. Nikpour S, Asgari AA. Colonoscopic evaluation of minimal rectal bleeding in average-risk patients for colorectal cancer. World Journal of Gastroenterology: WJG. 2008;14(42):6536.

104. Ogutu E, Okoth F, Lule G. Colonoscopic findings in Kenyan African patients. East African medical journal. 1998;75(9):540-3.

105. Oluyemi A, Odeghe E, Adeniyi O. Colonoscopy findings in lower gastrointestinal bleeding in Lagos: A comparative study based on age. Nigerian Journal of Clinical Practice. 2020;23(12):1656-9.

106. Orkin B, Smith L. Perineal manifestations of HIV infection. Diseases of the colon & rectum. 1992;35:310-4.

107. Parthasarathy N, Mangalore R, Liang V, et al., editors. Colonoscopy in octogenarians is a safe but overused investigation. JOURNAL OF GASTROENTEROLOGY AND HEPATOLOGY; 2015: WILEY-BLACKWELL 111 RIVER ST, HOBOKEN 07030-5774, NJ USA.

108. Pavone C, Caldarera E, Liberti P, et al. Correlation between chronic prostatitis syndrome and pelvic venous disease: a survey of 2,554 urologic outpatients. European urology. 2000;37(4):400-3.

109. Peery AF, Sandler RS, Galanko JA, et al. Risk factors for hemorrhoids on screening colonoscopy. PloS one. 2015;10(9):e0139100.

110. Petros JG, Bradley TM. Factors influencing postoperative urinary retention in patients undergoing surgery for benign anorectal disease. The American Journal of Surgery. 1990;159(4):374-6.

111. Poskus T, Buzinskienė D, Drasutiene G, et al. Haemorrhoids and anal fissures during pregnancy and after childbirth: a prospective cohort study. BJOG: An International Journal of Obstetrics & Gynaecology. 2014;121(13):1666-71.

112. Qin Y, Jiao C, Huang S, et al. " Zuoyuezi" dietary and behavioural associations with maternal health among puerperal women in South China. Asia Pacific journal of clinical nutrition. 2021;30(2):291-302.

113. Qiu X, Liu Y, Shi W, Lin G, Rong M, Wang B. Hemorrhoidal disease among doctors from grade-A tertiary hospitals in big cities of China: results from web-based doctors as patients survey. BMC gastroenterology. 2024;24(1):103.

114. Rai A, Mittal, S. Colonoscopic profile of lower gastrointestinal bleeding in children: A single centre experience from North India. Turkish Journal of Gastroenterology 2019-01-01;Volume 30(0):S817-S8

115. A RDMHSRM. Etiological Profile of Lower Gastrointestinal Bleed in a Tertiary Care Int J Pharm Clin Res. 2024;16(8): 1193–6.

116. Rhee JC, Lee KT. The causes and management of lower GI bleeding: a study based on clinical observations at Hanyang University Hospital. Gastroenterologia Japonica. 1991;26(Suppl 3):101-6.

117. Riss S, Weiser FA, Schwameis K, et al. The prevalence of hemorrhoids in adults. International journal of colorectal disease. 2012;27:215-20.

118. Robbins AJ, Lusczek E, Bellin MD, et al. Gastrointestinal Bleed After Total Pancreatectomy With Islet Autotransplant. Pancreas. 2021;50(6):841-6.

119. Romano L, Giuliani A, Paniccia F, et al. Sport practice and hemorrhoidal disease: results from a self-assessment questionnaire among athletes. International Journal of Colorectal Disease. 2025;40(1):1-6.

120. Rouhi M, Mohammed-alizadeh C S, Usefi H, Rouhi N. Postpartum morbidity and help-seeking behaviours in Iran. British Journal of Midwifery. 2011;19(3):178-84.

121. Sahu MK, Narayan, J., Uthansingh, K., Singh, A., Behera, M., Behera, D. The epidemiology of lower gastrointestinal bleed in Odisha. Indian Journal of Gastroenterology 2017;36(1):A30.

122. Sakr MA, Ebada HE, Abdelkader S, et al., editors. Outcome of colonoscopic screening in potential liver transplant candidates. Transplantation Proceedings; 2020: Elsevier.

123. Sanmee S, Supatrakul E, Sutharat P, Siriwittayakorn P. Colonoscopic Finding in 18-50 Years Old Adult with Rectal Bleeding. Journal of the Medical Association of Thailand. 2018;101(6).

124. Shafieipour S, Mohammadi E, Rukerd MRZ, et al. Gastrointestinal bleeding: prevalence, etiology, and outcomes in COVID-19 inpatients. Govaresh. 2023;28(1):30-5.

125. Sharma B, Sharma R, Bodh V, et al. Chronic Lower Gastrointestinal Bleeding: Etiological Profile and Role of Colonoscopy among Children from sub‑Himalayan Ranges of North India. Journal of Digestive Endoscopy. 2018;9(03):109-13.

126. Sharma S, Sharma, Y. P., Sood, S., Surya, M. Spectrum of gastrointestinal bleeding in pediatric population of hill state of north India. Indian Journal of Gastroenterology 2022;41(0):S162

127. Sheikh P, Régnier C, Goron F, Salmat G. The prevalence, characteristics and treatment of hemorrhoidal disease: results of an international web-based survey. Journal of comparative effectiveness research. 2020;9(17):1219-32.

128. Silva MA, León, G., Castillo, V., Hernández, N., Gordillo, B., Labastida, C. Hemorrhoids as a complication of pregnancy. Informe Medico 2009;11(4):225-35

129. Singh A, Singh M, Singh D. Health problems in rural elderly at Varanasi, Uttar Pradesh. The Journal of the Association of Physicians of India. 1996;44(8):540-3.

130. Tade A, Salami B, Musa A, Adeniji A. Anal complaints in Nigerians attending Olabisi Onabanjo University Teaching Hospital (OOUTH), Sagamu. Nigerian Postgraduate Medical Journal. 2004;11(3):218-20.

131. Tallarita T, Gurrieri C, Cappellani A, et al., editors. Clinical features of hemorrhoidal disease in renal transplant recipients. Transplantation proceedings; 2010: Elsevier.

132. Togo A, Kanté L, Poudiougo A, et al. Anal disorders in pregnant and postpartum women: epidemiological, diagnostic and therapeutic aspects in 10 maternities of Bamako in Mali. The Pan African Medical Journal. 2024;47:66.

133. Vardhan MV, Sahoo AK, Narayan J, et al. Lower Gastrointestinal Bleeding in Patients with Liver Cirrhosis: Prevalence, Outcomes, and Correlation with MELD Score in an Indian Tertiary Care Facility. Al-Rafidain Journal of Medical Sciences (ISSN 2789-3219). 2024;6(1):9-13.

134. Varma R, Mohammed NB, Rai AS. COLONOSCOPIC EVALUATION FOR LOWER GI BLEED AS SCREENING. Journal of Cardiovascular Disease Research. 2023;14(12):2084-98.

135. VECCHIA CL, PAGANO R, NEGRI E, DECARLI A. Smoking and prevalence of disease in the 1983 Italian National Health Survey. International journal of epidemiology. 1988;17(1):50-5.

136. La Vecchia C, Decarli A, Negri E, Ferraroni M, Pagano R. Height and the prevalence of chronic disease. Revue d'epidemiologie et de sante publique. 1992;40(1):6-14.

137. Yuhan R, Orsay C, DelPino A, et al. Anorectal disease in HIV-infected patients. Diseases of the colon & rectum. 1998;41:1367-70.

138. Zacharakis G, Almasoud A, Arahmaner O, et al. A 5-year evaluation of early-and late-onset sporadic colorectal cancer screening in central Saudi Arabia. Saudi Journal of Gastroenterology. 2023;29(2):95-101.

139. Zaman A, Hapke R, Flora K, Rosen H, Benner K. Prevalence of upper and lower gastrointestinal tract findings in liver transplant candidates undergoing screening endoscopic evaluation. Official journal of the American College of Gastroenterology| ACG. 1999;94(4):895-9.

140. Tan BK, Tsang CB, Nyam DC, Ho YH. Management of acute bleeding per rectum. Asian Journal of Surgery. 2004;27(1):32-8.

141. Wang T-F, Lee F-Y, Tsai Y-T, et al. Relationship of portal pressure, anorectal varices and hemorrhoids in cirrhotic patients. Journal of hepatology. 1992;15(1-2):170-3.

142. Haas PA, Haas GP, Schmaltz S, Fox TA. The prevalence of hemorrhoids. Diseases of the Colon & Rectum. 1983;26:435-9.

143. Mashbari H, Iskander O, Alyahyawi K, et al. Prevalence and risk factors of hemorrhoids in Jazan Region, Saudi Arabia: A cross-sectional study. Journal of Family Medicine and Primary Care. 2025;14(2):662-6.

144. Ciftel E, Ciftel S, Ciftel S, Mercantepe F, Akdogan RA. Hemorrhoidal Disease in the Diabetic Population: The Effects of Glucose Regulation and Lipid Profile. Life. 2025;15(2):178.

145. Alghareeb FY, Alghareeb SY, Issa HH, et al. Prevalence, Risk Factors, and Impact of Anal Disorders Following Sleeve Gastrectomy: A Single-Center Retrospective Study. Surgical Science. 2025;16(2):73-86.

146. Mahassadi AK, Motcheyo HC, Kouame DH, Yao-Bathaix FM. The Perception and Practices of Black African Subjects Toward Hemorrhoidal Disease: The Relevant Effects of Beliefs and Misconceptions in Côte d’Ivoire, West Africa. Research and Reports in Tropical Medicine. 2025:11-23.

147. Hepşen S, Üçgül E, Menekşe B, et al. Prevalence and risk factors of colon polyps and other colonic lesions in acromegaly: Insights from colonoscopy screening. Pituitary. 2025;28(2):1-8.

148. Contreras Aviles E, Philippe Ponce M, Guinea Lagunes A, et al. P0444 Frequency and Characteristics of Anorectal Pathologies in Patients with Inflammatory Bowel Disease. Journal of Crohn's and Colitis. 2025;19(Supplement_1):i953-i4.

149. Anderesen CK, Al-Najami I, Liu W, Orwoll E, Folkestad L. Risk of Gastrointestinal Diseases in Osteogenesis Imperfecta: A Nationwide, Register-Based Cohort Study. Calcified Tissue International. 2025;116(1):15.

150. Mutiibwa D, Turanzomwe S, Atukunda EC, Mugyenyi GR. Colonoscopy Findings at Divine Mercy Hospital-Father Bash Foundation, South-Western Uganda: A 6 Years Retrospective Analysis. Open Journal of Gastroenterology. 2025;15(3):91-100.
